# Tumoroid model recreates clinically relevant phenotypes of high grade serous ovarian cancer (HGSC) cells, carcinoma associated fibroblasts, and macrophages

**DOI:** 10.21203/rs.3.rs-6614892/v1

**Published:** 2025-06-19

**Authors:** Kathleen M. Burkhard, Ayush Semwal, Benjamin K. Johnson, Kristina C. Chu, Riley J. Kranick, Mihika Rayan, Analisa DiFeo, Hui Shen, Geeta Mehta

**Affiliations:** 1. Department of Biomedical Engineering, University of Michigan, Ann Arbor, MI.; 2. Department of Epigenetics, Van Andel Institute, Grand Rapids, MI.; 3. Department of Materials Science and Engineering, University of Michigan, Ann Arbor, MI.; 4. Department of Pathology, Michigan Medicine, University of Michigan, Ann Arbor, MI.; 5. Department of Obstetrics and Gynecology, Michigan Medicine, University of Michigan, Ann Arbor, MI.; 6. Macromolecular Science and Engineering, University of Michigan, Ann Arbor, MI.; 7. Rogel Cancer Center, University of Michigan, Ann Arbor, MI.; 8. Precision Health, University of Michigan, Ann Arbor, MI.

**Keywords:** organoids, tri-component tumoroids, spheroids, high grade serous ovarian cancer, tumor microenvironment, carcinoma-associated mesenchymal stem cells (MSC) (CA-MSC), tumor associated macrophages (TAM), alternately activated macrophages, molecular subtyping, scRNA-seq

## Abstract

Ovarian cancer, the gynecological malignancy with the lowest survival rate, is significantly influenced by the tumor microenvironment. The mesenchymal subtype of high-grade serous carcinoma (HGSC) shows poor outcomes due to high stromal and low immune response. Single-cell RNA sequencing (scRNA-seq) of HGSC metastatic ascites has identified carcinoma-associated fibroblasts (CAFs), macrophages, and carcinoma-associated mesenchymal stem cells (CA-MSCs) as crucial drivers of immune exclusion, chemotherapy resistance, metastasis, and stem-like cell propagation. To explore this complex signaling, we developed heterogeneous tri-component tumoroids, incorporating HGSC cells (OVCAR3, OVCAR4, OVCAR8), primary MSCs, and U937-derived M2-like macrophages (M2-AAM) in defined ratios, each labeled with a fluorescent protein for distinct analysis. Upon a 48-hour treatment with carboplatin and/or paclitaxel, HGSC cells in tri-component tumoroids exhibited higher chemoresistance than HGSC-only spheroids. Flow cytometry revealed significant increases in cancer stem-like cell (CSC) markers CD44 and CD90 in the tri-component tumoroids. Conditioned medium from the tri-component tumoroids significantly enhanced HGSC cell migration compared to spheroids. Invasion assays further demonstrated that tri-component tumoroids penetrated monolayer of mCherry-labeled LP-9 mesothelial cells more effectively than spheroids. Additionally, scRNA-seq of tri-component tumoroids identified a unique cancer cell cluster enriched in epithelial-mesenchymal transition (EMT) and matrisome signatures, featuring a 14-gene signature linked to poor survival. MSCs in these tri-component tumoroids displayed a myofibroblastic-CAF signature, while macrophages indicated an ECM-associated and immunosuppressive phenotype. In conclusion, our 3D heterogenous tri-component tumoroids replicate key HGSC phenotypes, such as chemoresistance, CSC enrichment, migration, invasion, and EMT. This platform is invaluable for studying HGSC microenvironment interactions and preclinical testing of targeted therapies.

## Introduction

Ovarian cancer is the most lethal gynecological malignancy.^[1]^ Notably, a recent long-term randomized controlled trial demonstrated that early detection of high grade serous ovarian cancers (HGSC) does not provide survival benefits.^[2]^ Additionally, non-cancerous stromal and immune cells are present in the tumor mass and ascites of ovarian cancer.^[3–7]^ In 2008, Tothill et al. identified four molecular subtypes of HGSC which correlated with survival.^[8]^ Later, The Cancer Genome Atlas (TCGA) outlined similar subtypes: Differentiated, Immunoreactive, Mesenchymal, and Proliferative.^[9]^ The mesenchymal molecular subtype was characterized by high extracellular matrix protein expression and contribution of stromal cells, such as myofibroblasts, with the worst survival. Further studies confirmed this subtype classification, including PROVAR and CLOVAR, and suggested the subtypes could overlap and were not mutually exclusive.^[10–12]^ Molecular subtype classifications have varied, with some studies having identified two or three subtypes, highlighting the heterogeneity across race, ethnicity, and genomic instability.^[13–15]^ Even though molecular subtype signatures have not yet been clinically adopted in HGSC, they demonstrate robust evidence of prognostication of chemoresistance and tumor recurrence.^[8–10,12,16–18]^

Recent single-cell analyses of HGSC by various groups have identified stromal carcinoma associated fibroblasts (CAFs) and macrophages as major constituents of the tumor microenvironment.^[5,19–21]^ Several studies have investigated the contribution of carcinoma associated MSCs (CA-MSCs) and CAFs on HGSC phenotype. CA-MSC have been shown to be drivers of immune exclusion, resistance to chemotherapy, metastatic potential, and increased stem-like cells via PDGF-BB, BMP4 and the Hedgehog pathway.^[3,4,7]^ Similarly, stromal macrophages have been shown to adopt an alternatively activated M2-like phenotype (M2-AAM) in co-culture with HGSC cells.^[22–24]^ Furthermore, the reciprocal relationships between cancer cells and the M2-AAM enhance a stem-like, chemoresistant, and metastatic phenotype in HGSC.^[3,25–28]^ These studies have revealed critical interactions between stromal cells and tumor cells. However, the multi-directional signaling between multiple stromal cells as well as the combined effect of more than one stromal cell type on HGSC has not been widely studied. Due to the influence of non-cancerous supporting cells in the tumor on cancer cells, it is important to include them in the *in vitro* mechanistic models of HGSC. One strategy to accomplish this is by using patient-derived organoids (PDOs). PDOs are generated by dissociating patient tumor tissue into single cells and suspending them in an ECM-mimic, often Matrigel, where they self-assemble into 3D organoids.^[29,30]^ The tumor genetic profile, morphology, and drug response are recapitulated by these PDO models much better than by 2D cell culture.^[30–34]^ However, studying the impact of individual cell populations using PDOs is challenging since organoids are initiated by a heterogeneous population of patient-derived cells, which is not well defined and can vary between patients and tumor samples.^[5,30,34]^ Furthermore, some cell types can be lost over time in organoid culture as epithelial tumor cells predominate.^[29,30,34]^

To address these challenges, in this work we developed a 3D tri-component tumoroid platform incorporating HGSC cells, mesenchymal stem cells (MSC), and macrophages (M2-AAM) in defined ratios and numbers. This platform allows for the study of multi-directional cell-cell communications in a controlled high-throughput setting. We characterized the contributions of stromal cells (MSC and M2-AAM) to the migration, invasion, chemotherapy response, and stemness of the HGSC. We further characterized the multi-directional signaling by single-cell RNA sequencing (scRNA-seq) and identified a unique cluster of HGSC only in the tri-component tumoroids, with an elevated EMT signature that is clinically significant for overall survival. Our findings underscore the clinical significance of these multi-directional stromal interactions.

By developing and characterizing physiologic ovarian cancer tri-component tumoroid platforms that feature multiple cell types (HGSC, carcinoma associated mesenchymal stem cells, and macrophages), we can address fundamental questions about tumor heterogeneity, immune plasticity and immune modulation. This work paves the way for improving long-term cure rates in ovarian cancers and inspires new engineering strategies to target stromal cell phenotypes, breaking the trophic interactions in HGSC and other epithelial cancers.

## Results

### Characterization of tumoroids with fluorescently labeled HGSC, MSC, and M2.

**Figure 1:**
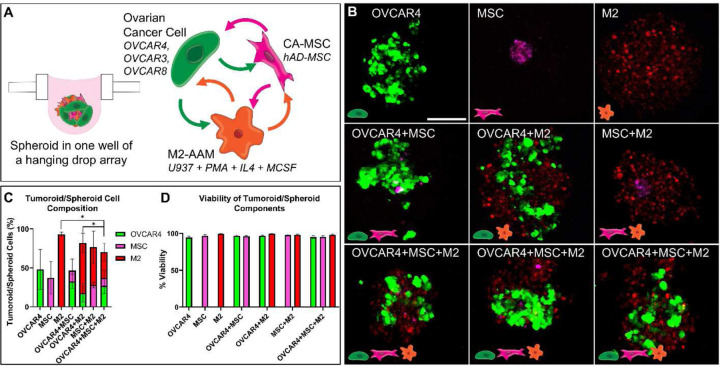
HGSC 3D tumoroids are composed of HGSC cells, CA-MSC and M2-AAM. Tri-component tumoroids are composed of viable HGSC cells, MSC and M2-AAM. **A)** Cells were transduced with distinct fluorescent proteins, with OVCAR3,4,8 expressing GFP, MSC expressing smURFP, and M2 expressing mCherry. Tumoroids were generated with 600 cells of OVCAR4, MSC, and/or M2, and were maintained for 5 days, then analyzed and sorted using fluorescence-activated cell sorting (FACS). **B)** Representative images of fluorescent tumoroids of various composition were obtained using confocal microscopy. Scale bar = 200 µm. **C)** The percentage of cells expressing each fluorescent protein was quantified using FACS to determine the percentage of each cell type in the tumoroid. Repeated measures one-way ANOVA with Geisser-Greenhouse correction and Tukey’s multiple comparisons was performed. The percentage of OVCAR4 cells in OVCAR4+M2 is significantly different than in OVCAR4+MSC+M2 tri-component tumoroids (p = 0.0481, *p<0.05). The percentage of M2 in M2 spheroids is significantly different than in OVCAR4+MSC+M2 tri-component tumoroids (p = 0.0153, *p<0.05). **D)** The viability of each cell type was quantified by analyzing the percentage of live DAPI-negative cells (i.e., viable cells) within each fluorescent cell population. The differences in cell viability between conditions is not statistically significant.

In order to model the cell-cell interactions within the ovarian tumor microenvironments, we created tri-component tumoroids by utilizing HGSC cell lines (OVCAR3, OVCAR4, and OVCAR8),^[35]^ human adipose-derived MSC to represent CA-MSC, and M2-AAM to represent TAM.^[22]^ Each of these cell types was labeled with a distinct fluorescent protein to enable separate visualization and analysis. HSGC cell lines were labeled with GFP using lentiviral transduction while MSC and U937 were labeled with smURFP and mCherry, respectively. M2 were derived by treating mCherry-labeled U937 with 5 ng/mL PMA for 24 h followed by 200 ng/mL IL4 and MCSF for 48 h. Tumoroids or spheroids were created with these fluorescently labeled cells by seeding 600 cells/well of each cell type in a hanging drop plate for the following conditions: OVCAR4, MSC, M2, OVCAR4+MSC, OVCAR4+M2, MSC+M2, and OVCAR4+MSC+M2 (Fig. 1A, Supplementary Table S1). After 5 days in the spheroids or tumoroids, confocal microscopy was conducted to reveal that each cell type was present via their respective fluorescent protein (Fig. 1B). Tumoroids or spheroids were dissociated into single cell suspensions and analyzed using flow cytometry to further confirm that each cell type was present (Fig. 1C). Finally, flow cytometry with DAPI verified that each cell type remained viable in the spheroids, with the percentage of viable cells greater than 94% in all conditions (Fig. 1D). Therefore, each of the three cell types remained present and viable in the tri-component tumoroids and spheroids after 5 days, confirming that this platform can be used to study their cell-cell interactions in subsequent analyses.

### Increased chemoresistance and stemness in tumoroids.

**Figure 2:**
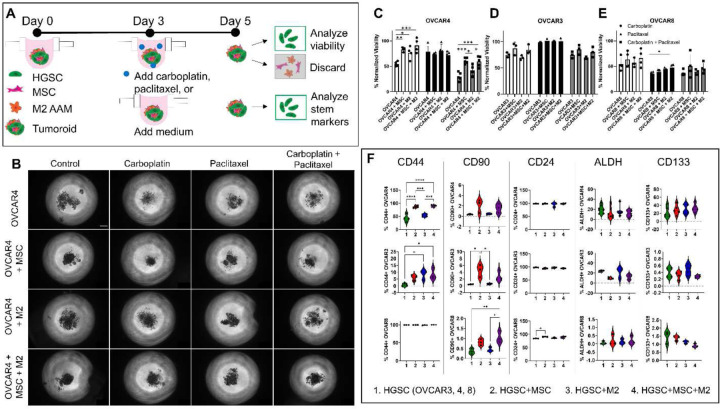
Tri-component tumoroids are more resistant to chemotherapy and express more cancer stem-like cell markers. **A)** Tumoroids were generated with 600 cell per well of each cell type. On day 3, spheroids were treated with 300 µM carboplatin and/or 10 µM paclitaxel and incubated for 48 hours. On day 5, tumoroids were collected and analyzed using flow cytometry to determine the percentage of live (DAPI-negative) cells within the cancer cell population (GFP+). **B)** Representative bright field images of OVCAR4 tumoroids on day 5 after 48 hours of treatment with drug or vehicle control. Scale bar = 200 µm. Viability of the GFP-labeled HGSC cells in response to carboplatin (white), paclitaxel (black), and carboplatin with paclitaxel (grey) was quantified using flow cytometry for DAPI exclusion and normalized to the vehicle control viability for **C)** OVCAR4, **D)** OVCAR3, and **E)** OVCAR8 spheroids. **F)** The expression of cancer stem-like cell markers CD44, CD90, CD24, ALDH, and CD133 on control spheroids and tumoroids was analyzed on day 5 using flow cytometry. Ordinary one-way ANOVA with Tukey’s multiple comparisons was performed. * p ≤ 0.05, ** p ≤ 0.01, *** p ≤ 0.001, **** p ≤ 0.0001.

Tumor recurrence and resistance to chemotherapy remain a major challenge in treatment of HGSC. Given that platinum and taxol are the standard chemotherapy of care in HGSC in the neoadjuvant or adjuvant setting,^[36–38]^ we chose these agents to assess chemoresistance in the tri-component tumoroids. In order to separate the responses of HGSC from those of MSC or M2, spheroids or tumoroids were created using GFP-labeled OVCAR3, OVCAR4, or OVCAR8. On day 3, spheroids or tumoroids were treated with 300 µM carboplatin, 10 µM paclitaxel, or a combination of 300 µM carboplatin and 10 µM paclitaxel for 48 h. On day 5, spheroids were collected and dissociated to analyze viability of GFP-labeled ovarian cancer cells with DAPI using flow cytometry (Fig. 2A). Representative brightfield images displayed more debris and smaller tumoroids or spheroids in drug treated conditions, highlighting cell death (Fig. 2B, Supplementary Figure S1). OVCAR4 were significantly more resistant to carboplatin in OVCAR4+MSC, OVCAR4+M2, and OVCAR4+MSC+M2 tumoroids compared to OVCAR4 spheroids, with 83.4% ± 2.07, 76.8% ± 6.17, 91.3% ± 6.94, and 54.7% ± 2.55 viability normalized to vehicle control spheroids, respectively (Fig. 2C). All four tumoroid compositions of OVCAR4 were less sensitive to paclitaxel with normalized viability ranging from 72.0% to 80.4% and did not show significant differences in viability between conditions. When treated with a combination of carboplatin and paclitaxel, there was a similar trend to the carboplatin treatment, with higher viability in OVCAR4+MSC, OVCAR4+M2, and OVCAR4+MSC+M2 tumoroids compared to OVCAR4 spheroids with 60.9% ± 2.62, 42.0% ± 4.44, 59.9% ± 4.66, and 30.0% ± 3.69 normalized viability; however, only OVCAR4+MSC and OVCAR4+MSC+M2 tumoroids were significantly higher compared to OVCAR4 spheroids (Fig. 2C, Supplementary Figure S2). OVCAR3 showed similar trends to OVCAR4, with higher viability in OVCAR3+MSC and OVCAR3+MSC+M2 tumoroids compared to OVCAR3 spheroids treated with carboplatin, or carboplatin and paclitaxel together with normalized viability for carboplatin of 87.7% ± 6.79, 84.8% ± 5.17, and 75.3% ± 2.67, respectively and normalized viability for carboplatin with paclitaxel of 85.0% ± 5.37, 80.1% ± 5.75, and 75.1% ± 3.62, respectively. OVCAR3 were the most resistant to paclitaxel with normalized viability ranging between 99.3% and 102.1% and did not show a difference in viability between tumoroid conditions (Fig. 2D, Supplementary Figure S3). Finally, similar trends were again observed in OVCAR8. OVCAR8+MSC and OVCAR8+MSC+M2 tumoroids had higher viability compared to OVCAR8 spheroids treated with carboplatin (63.8% ± 7.85, 66.2% ± 8.62, and 54.3% ± 6.58, respectively) or carboplatin and paclitaxel together (50.4% ± 11.45, 46.1% ± 5.18, and 37.1% ± 2.87, respectively). However, OVCAR8 was the most sensitive to paclitaxel, which is consistent with literature,^[39]^ and OVCAR8+MSC+M2 tri-component tumoroids were significantly more resistant compared to OVCAR8 spheroids with normalized viability of 47.2% ± 3.55 and 35.8% ± 1.99, respectively (Fig. 2E, Supplementary Figure S4).

Given that the emergence of chemoresistance is linked to increased cancer stem-like characteristics, we quantified the following markers of HGSC cancer stem-like cells:^[40–43]^ CD44,^[44–46]^ CD24,^[47–49]^ CD90,^[50–52]^ CD133,^[53–55]^ and ALDH activity.^[54,56–59]^ Markers of cancer stem-like cells on GFP-labeled cancer cells were evaluated using flow cytometry after harvesting tumoroids. CD44 expression was increased in OVCAR4+MSC, OVCAR4+M2, and OVCAR4+MSC+M2 tumoroids compared to OVCAR4 spheroids with 86.2±1.92%, 54.1±2.55%, 89.6±1.78%, and 43.2±6.83%, respectively, although OVCAR4+M2 (54.1±2.55%) was not significantly higher than OVCAR4 (43.2±6.83%) (Fig. 2F). CD44 expression was also increased in OVCAR3+MSC, OVCAR3+M2 and OVCAR3+MSC+M2 tumoroids compared to OVCAR3 spheroids with 6.0±1.18%, 8.4±2.01%, 8.5±2.35%, and 0.6±0.61% respectively, although OVCAR3+MSC was not significantly higher than OVCAR3 (Fig. 2F). All spheroids and tumoroids generated with OVCAR8 maintained a high baseline fraction of CD44 positive cancer cells (e.g., 99.7±0.07% for OVCAR8-only spheroids). Similar trends were observed in CD90 expression, with OVCAR8+MSC+M2 and OVCAR8+MSC tumoroids demonstrating significantly higher CD90 expression compared to OVCAR8-only spheroids (Fig. 2F, Supplementary Figure S5). The OVCAR4+MSC+M2 and OVCAR4+MSC tumoroids also revealed significantly higher CD90 positive cells compared to OVCAR4-only spheroids (2.06±0.74%, 1.52±0.51%, and 0.38±0.05% for OVCAR4+MSC, OVCAR4+MSC+M2, and OVCAR4, respectively) (Fig. 2F). In summary, increased resistance to carboplatin, paclitaxel, and combination treatment was observed in the tri-component tumoroids compared to HSGC-only spheroids, with MSC contributing more to the CSC-like phenotype than macrophages. Therefore, the increased chemoresistance observed in the HGSC tumoroids is consistent with increased expression of CSC markers CD44 and CD90 in the tumoroids compared to spheroids.^[60]^

### Increased migration from secreted factors in tumoroids.

**Figure 3:**
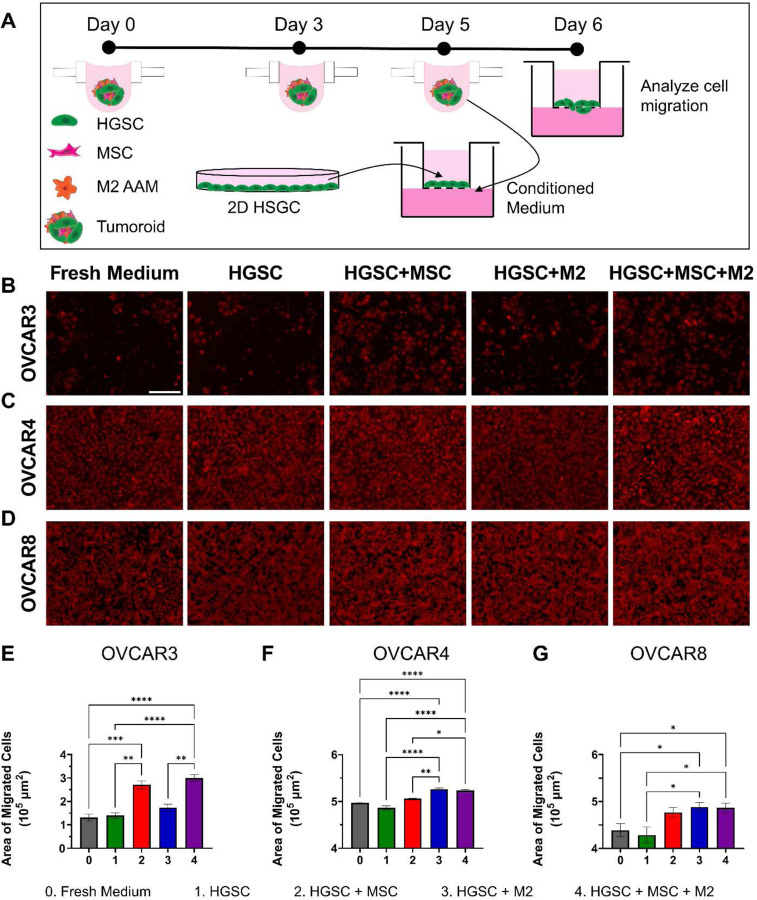
Conditioned medium from tumoroids containing MSC and M2 induce more migration in HGSC cells. **A)** Conditioned medium from tumoroids on day 5 was placed in the bottom chamber of the transwell, with HGSC cells in starved medium in the top. Cells migrated for 24 hours, then membranes were fixed and stained with crystal violet and cells were removed from the top of the membrane. **B-D)** Representative fluorescent images of transwell membranes with crystal-violet labeled migrated HGSC cells were obtained for **B)** OVCAR3, **C)** OVCAR4, and **D)** OVCAR8 cell lines. Scale bar = 200 µm. **E-G)** The area of the membrane covered by migrating cells was analyzed using ImageJ for **E)** OVCAR3, **F)** OVCAR4, and **G)** OVCAR8 HGSC cells within the tumoroids. Kruskal-Wallis with Dunn’s multiple comparisons tests were performed. * p ≤ 0.05, ** p ≤ 0.01, *** p ≤ 0.001, **** p ≤ 0.0001.

Approximately 60% of patients already have metastatic lesions in the peritoneal cavity at diagnosis which is a major challenge to the treatment of ovarian cancer due to decreased efficacy of debulking surgery and chemotherapy.^[61]^ During metastasis, a cancer cell must detach from the tumor, migrate to the metastatic site, then adhere and invade into the tissue of the secondary site.^[62]^ Given that the multi-directional and reciprocal paracrine signaling between MSC, M2 and HGSC cells can influence migration of cancer cells, we sought to quantify the impact of soluble signals only on the migratory potential of HGSC due to secreted factors synthesized by MSC and M2 via a transwell assay using conditioned medium from the spheroids and tumoroids. Spheroids were generated in hanging drop plates and after 5 days, the collected conditioned medium was placed in the bottom of a well. Matching HGSC were collected from cell culture plates and placed in the top of the transwell insert in starved medium. Cells were allowed to migrate for 24 hours, then transwell membranes were fixed and stained with crystal violet (Fig. 3A). Cells were removed from the top of the membrane and membranes were imaged to quantify the area covered by cells that migrated through the membrane (Fig. 3B-D). Significantly more OVCAR3 cells migrated when stimulated with conditioned medium from OVCAR3+MSC (2.71 x 10^6^ ± 1.73 x 10^5^ µm^2^; 1.94 fold) and OVCAR3+MSC+M2 (3.00 x 10^6^ ± 1.48 x 10^5^ µm^2^; 2.14 fold) tumoroids compared to OVCAR3-only spheroids (1.40 x 10^6^ ± 1.10 x 10^5^ µm^2^) or fresh medium (1.31 x 10^6^ ± 1.51 x 10^5^ µm^2^; 0.94 fold) (Fig. 3E). OVCAR4 were more migratory at baseline, covering 86.0% of the membrane in the OVCAR4-only spheroid control compared to OVCAR3, which covered 24.7% of the membrane in the OVCAR3-only spheroid control. Additionally, more migration of OVCAR4 was observed with conditioned medium from tumoroids containing macrophages. Significantly more OVCAR4 migrated with conditioned medium from OVCAR4+M2 (5.26 x 10^6^ ± 2.52 x 10^4^ µm^2^; 1.08 fold) and OVCAR4+MSC+M2 (5.23 x 10^6^ ± 2.27 x 10^4^ µm^2^; 1.08 fold) tumoroids compared to OVCAR4 spheroids (4.87 x 10^6^ ± 4.35 x 10^4^ µm^2^) or fresh medium (4.97 x 10^6^ ± 1.13 x 10^4^ µm^2^; 1.02 fold) (Fig. 3F). OVCAR8 also had a high baseline migratory potential, with 75.7% of the membrane being covered by migrated cells in the OVCAR8-only spheroid control. Significantly more OVCAR8 cells migrated with conditioned medium from OVCAR8+MSC (4.77 x 10^6^ ± 1.08 x 10^5^ µm^2^; 1.11 fold), OVCAR8+M2 (4.88 x 10^6^ ± 9.16 x 10^4^ µm^2^; 1.14 fold), and OVCAR8+MSC+M2 (4.86 x 10^6^ ± 9.89 x 10^4^ µm^2^; 1.14 fold) tumoroids compared to OVCAR8 spheroids (4.28 x 10^6^ ± 1.73 x 10^5^ µm^2^) or fresh medium (4.39 x 10^6^ ± 1.39 x 10^5^ µm^2^; 1.02 fold) (Fig. 3G). OVCAR8 displayed increased migration when exposed to conditioned medium from tumoroids containing MSC, M2, or both MSC and M2. Conversely, OVCAR4 showed enhanced migration in response to M2, but not MSC, and OVCAR3 showed enhanced migration in response to MSC, but not M2. Despite differences in cell migration between cell lines, the conditioned medium from the tri-component tumoroids resulted in significantly more migration of all three HGSC cell lines (OVCAR3, OVCAR4, and OVCAR8) compared to spheroid controls.

### Increased invasion of tumoroids through LP-9 monolayer.

**Figure 4:**
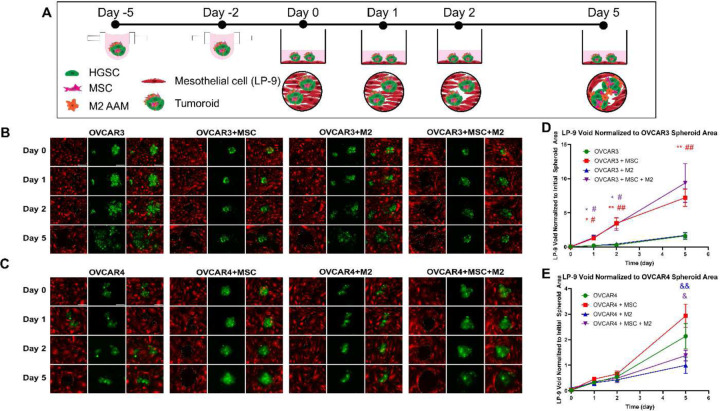
Co- and tri-component tumoroids have increased ability to invade through mesothelial cell monolayer. **A)** Day 5 tumoroids were transferred to a confluent monolayer of mCherry-labeled LP-9 mesothelial cells. Fluorescent images of the monolayer and GFP-labeled HGSC cells in the tumoroids (or spheroids) were acquired on days 0, 1, 2, and 5. **B-C)** Representative images of mesothelial monolayer and tumoroids or spheroids from **B)** OVCAR3 and **C)** OVCAR4 HGSC cells. Scale bar = 200 µm. **D-E)** The void area in the mesothelial monolayer was quantified using ImageJ and normalized to the initial tumoroids or spheroid area and graphed over time for **D)** OVCAR3 and **E)** OVCAR4. A mixed-effects model with Geisser-Greenhouse correction and Tukey’s multiple comparisons was performed. * indicates comparisons significantly different than OVCAR3, # indicate comparisons significantly different than OVCAR3+M2, and & indicates comparisons significantly different than OVCAR4+MSC. * p ≤ 0.05, ** p ≤ 0.01, *** p ≤ 0.001, **** p ≤ 0.0001.

Primary ovarian cancer cells detach and disseminate intraabdominally to sites of metastases, particularly the omentum, peritoneum, and the bowel, which are lined by mesothelial cells.^[63]^ In order for ovarian cancer cells to efficiently form metastases, they need to clear the cellular and mechanical barrier formed by mesothelial cells. Therefore, we utilized mesothelial cell layer clearing as a functional phenotype of HGSC invasion in the co- and tri-component tumoroids.^[64]^ The invasion potential of tumoroids was evaluated by their ability to clear though a monolayer of mesothelial LP-9 cells. After 5 days in hanging drops, tumoroids and spheroids with GFP-labeled HGSC cells were collected and plated onto a monolayer of mCherry-labeled LP-9 cells (Fig. 4A). Images of the LP-9 monolayer and GFP-containing tumoroids or spheroids were acquired at 2 h, 24 h, 28 h, and 120 h after adding the GFP-labeled tumoroids or spheroids (Fig. 4B, 4C). The area of the void in the LP-9 monolayer was measured and normalized to the initial tumoroid or spheroid area. OVCAR3+MSC (Day 1: 1.26 ± 0.305, Day 2: 3.47 ± 0.799, Day 5: 7.21 ± 1.27) and OVCAR3+MSC+M2 tumoroids (Day 1: 1.37 ± 0.345, Day 2: 3.32 ± 0.889, Day 5: 9.36 ± 2.83) invaded through the LP-9 monolayer significantly more than OVCAR3-only spheroids (Day 1: 0.193 ± 0.051, Day 2: 0.294 ± 0.076, Day 5: 1.62 ± 0.509) and OVCAR3+M2 tumoroids (Day 1: 0.192 ± 0.047, Day 2: 0.437 ± 0.096, Day 5: 1.69 ± 0.369) (Fig. 4B). OVCAR4 spheroids (Day 5: 2.13 ± 0.496) and OVCAR4+MSC tumoroids (Day 5: 2.93 ± 0.454) invaded the most, significantly more than OVCAR4+M2 (Day 5: 0.996 ± 0.317) and OVCAR4+MSC+M2 tumoroids (Day 5: 1.38 ± 0.208) (Fig. 4C). Interestingly, OVCAR3 were more invasive when MSC were also incorporated into the tumoroids, whereas the trend was not as drastic in the OVCAR4 conditions (Fig. 4D). OVCAR4-only spheroids displayed slightly more baseline invasion into the LP-9 monolayer compared to OVCAR3-only spheroids, which is consistent with OVCAR4 displaying more migration in the transwell assay (Fig. 4E and Fig. 3F). In both cell lines (OVCAR4 and OVCAR3), the presence of M2 did not impact invasion into LP-9 monolayer, whereas MSC increased tumoroid or spheroid invasion, highlighting the importance of juxtacrine signaling between MSC and invading HGSC.

### Cell-cell signaling in tumoroids.

**Figure 5:**
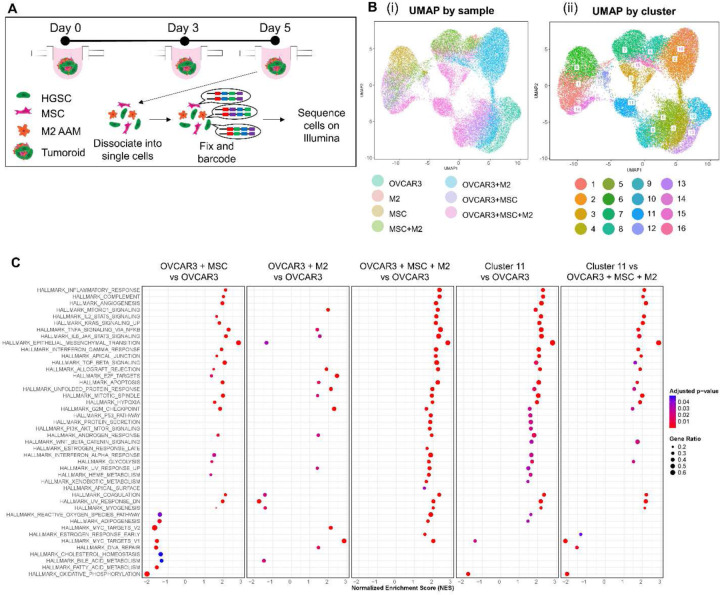
Single cell sequencing reveals upregulation of EMT and other cancer-associated pathways in tri-component tumoroids. **A)** Tumoroids were generated with 600 cells/well of each cell type (HGSC cell line OVCAR3, human adipose derived mesenchymal stem cells (MSC), alternately activated macrophages derived from U937 cell line (M2-AAM)). On day 5, tumoroids were collected, re-suspended into single cells, and sequenced via Parse Bioscience whole transcriptome kit. **B)** Uniform manifold approximation and projection (UMAP) plot of all sequenced single cells from tri-component tumoroids. Colors indicate sample identity as noted on the top of the plot (3 individual cell types: HGSC, MSC, M2-AAM, 3 co-tumoroids or co-spheroids: HGSC+MSC, HGSC+M2-AAM, MSC+M2-AAM, 1 tri-component tumoroid: HGSC+MSC+M2-AAM). **C)** GSEA hallmark signaling pathways that are activated in the HGSC tumor cells (OVCAR3), stromal mesenchymal stem cells (MSC), and macrophages (M2-AAM) compared to their respective single cell type control (OVCAR3, MSC, M2-AAM) from the scRNA-seq analysis of tri-component tumoroids and their mono-spheroid controls). EMT, Inflammatory, Interferon-α, Interferon-γ signaling are activated in the HGSC tumor cells in the tri-component tumoroids. Meanwhile, CA-MSC undergo activation of WNT/β-catenin, mTORC1, Hedgehog, and TGF-β signaling in the tri-component tumoroids. The M2-AAM also activate Interferon-α, EMT, TGF-β, Interferon-γ, Inflammatory and mTORC1 signaling in the tri-component tumoroids.

Thus far, different phenotypes of chemotherapy response, stemness, migration, and invasion, were observed due to the incorporation of different stromal cells in the tumoroids. Therefore, we next investigated the cellular signaling behind these differences using scRNA-seq. Tumoroids and spheroids were collected on day 5 for single-cell RNA sequencing (Fig. 5A), and doublets and low library counts were filtered out and the remaining cells were separated into 16 clusters. Three intriguing clusters were identified that localized near one of the main cell types (i.e., HGSC, MSC, or M2), but more toward the middle of the UMAP plot (Fig. 5B). ***Cluster 3*** was grouped near the other M2 clusters and contained cells mostly labeled with GFP or mCherry, and a few with smURFP. Cells in ***Cluster 11*** were congregated near the other OVCAR3 clusters and contained cells mostly labeled with GFP and a few with smURFP. Lastly, cells in ***Cluster 14*** grouped near the other MSC clusters and contained cells mostly labeled with smURFP and a few with GFP. The overlapping fluorescent proteins in these clusters indicated potential fusion of two or more cell types, an observation made in breast, pancreatic and intestinal cancers, where cancer cells and MSC or cancer cells and macrophages were fused together and contributed to enhanced metastases.^[65–70]^

After removal of the overlapping cells, Gene Set Enrichment Analysis (GSEA) was performed to compare OVCAR3 from OVCAR3+MSC+M2 tri-component tumoroids to OVCAR3 spheroids, and to compare ***Cluster 11*** to OVCAR3 from OVCAR3+MSC+M2 tumoroids and OVCAR3 spheroids (Fig. 5C). The epithelial mesenchymal transition (EMT) hallmark was the most enriched gene set in OVCAR3 from OVCAR3+MSC+M2 tumoroids compared to OVCAR3 spheroids, as well as in OVCAR3+MSC tumoroids and ***Cluster 11*** compared to OVCAR3 spheroids. EMT was further enriched in ***Cluster 11*** compared to OVCAR3 from OVCAR3+MSC+M2 tumoroids. The TNFα signaling via NFκB and IL6-JAK-STAT3 gene sets were also both highly enriched, with each tumoroid comparison showing activation of these pathways. The TGFβ signaling gene set was also highly enriched in these tumoroid comparisons, with the exception of OVCAR3+M2 versus OVCAR3. Interestingly, a few gene sets were enriched in tri-component OVCAR3 or ***Cluster 11*** only, and not in the other tumoroid conditions, including p53 pathway, protein secretion, PI3K/AKT/mTOR signaling, WNT-β-catenin signaling, estrogen response late, xenobiotic metabolism, and apical surface hallmark gene sets (Fig. 5C).

**Figure 6:**
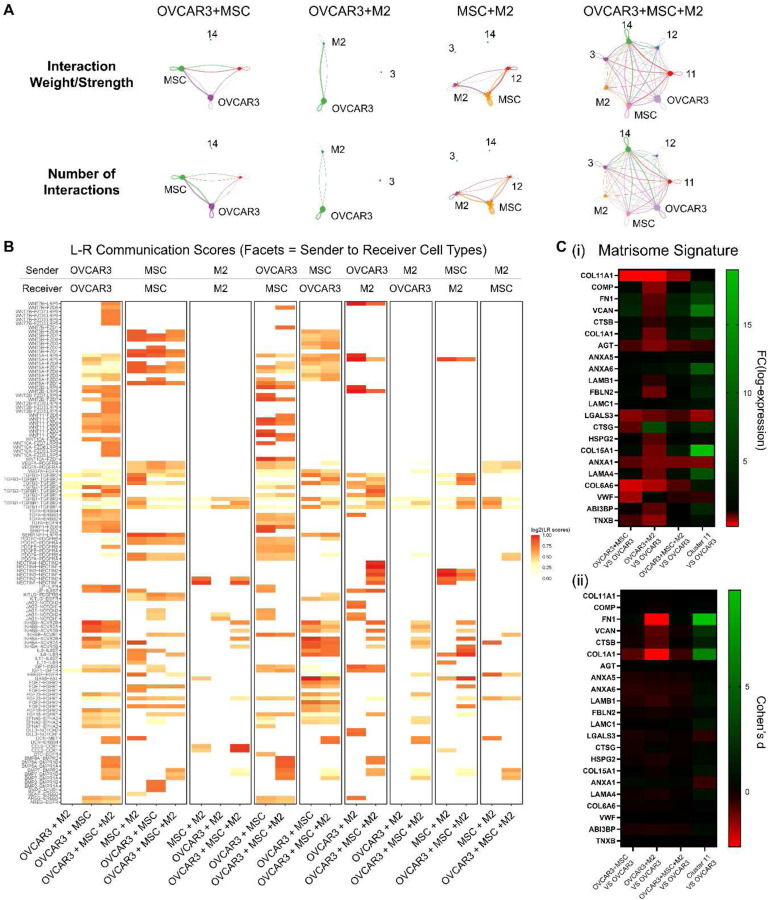
OVCAR3, MSC, and M2 communicate through pathways related to inflammation, proliferation, and metastasis, including Wnt. Gene expression data was analyzed using *CellChat* to identify ligand-receptor interaction between cell types. **A)** Cell interaction plots displaying the number and strength of interactions between each cell type is displayed for the three co-component tumoroids and the tri-component tumoroids. **B)** A heatmap of the ligand-receptor pairs between tumoroid condition reveals Wnt and TGF-β signaling from MSC and M2-AAM to OVCAR3 cells. **C)** Comparing the Matrisome signature from Pearce et al. to the tri-component tumoroids revealed an increased expression of the Matrisome in the tri-component OVCAR3 and ***Cluster 11*** cells as calculated by (i) fold change in gene log-expression and (ii) Cohen’s d effect size.^[73]^

In order to understand how these gene sets were being enriched in the OVCAR3 cells, the multi-directional signaling between OVCAR3, MSC, and M2 were investigated using the *CellChat* and *CellCall* databases.^[71,72]^ *CellChat* revealed that each cell type present in a given tumoroid condition interacted with every other cell type, including itself, albeit at varying levels of interaction and strength (Fig. 6A). These databases calculated the probability of ligand and receptor binding between sender and receiver cells. Using the *CellCall* database, several Wnt ligands appeared in the cell-cell interactions in the tri-component tumoroids, which is consistent with the Wnt/β-catenin signaling pathway being enriched in GSEA (Fig. 6B and Fig. 5C). WNT5B and WNT5A from MSC were found to bind to FZD receptors on OVCAR3 in OVCAR3+MSC+M2 tri-component tumoroids. Additionally, in these tri-component tumoroids, WNT5A and WNT5B from MSC bound to FZD receptors on MSC in an autocrine manner. WNT5A from MSC bound to LRP5 on M2 and WNT5A from OVCAR3 bound to FZD receptors on MSC, as well as on OVCAR3 in an autocrine manner. Furthermore, additional WNT ligands sent from OVCAR3 bound to MSC, M2, and OVCAR3. Quantitative PCR confirmed that MSC expressed WNT5A and WNT5B at higher levels than any other WNT ligands (Supplementary Figure S6). The TGF beta signaling hallmark gene set was also enriched in ***Cluster 11*** compared to OVCAR3 from OVCAR3+MSC+M2 tumoroids and OVCAR3-only spheroids. Analysis using *CellCall* revealed TGFB1 from M2 bound to TGFRB1 and TGFRB2 on OVCAR3, and INHBB and INHBA from M2 bound to ACVR2A, ACVR1B, and ACVR2B on OVCAR3 (Fig. 6B). These ligand receptor interactions were also observed in M2-M2, MSC-M2, MSC-OVCAR3, OVCAR3-M2, and OVCAR3-OVCAR3 signaling pairs.

Given the increased EMT in ***Cluster 11***, the tumor metastatic matrisome signature from Pearce, et al. was examined in OVCAR3 across the different co- and tri-component tumoroids.^[73]^ OVCAR3+M2 did not indicate upregulation of the matrisome signature; however, OVCAR3+MSC, OVCAR3+MSC+M2, and ***Cluster 11*** revealed its significant activation, with ***Cluster 11*** showing the most upregulation of the matrisome signature. *FN1*, *VCAN*, *CTSB*, and *COL1A1* were the most prominently upregulated genes in this signature, with fold change log-expression values of 7.28, 12.06, 2.94, and 5.96, respectively, and Cohen’s d values of 9.58, 2.33, 1.38, and 6.57, respectively, for ***Cluster 11*** vs OVCAR3 (Fig. 6C). Overall, many ligand-receptor interactions were identified in the tumoroids and the tri-component tumoroids had the most complex signaling due to the presence of stromal and immune cell types. EMT, Wnt signaling, and other signaling pathways associated with progression of HGSC were observed to be enriched in the tri-component tumoroids compared to OVCAR3-only spheroids.

### Myofibroblastic CAF and ECM-associated and immunosuppressive macrophage phenotype in tumoroids.

**Figure 7:**
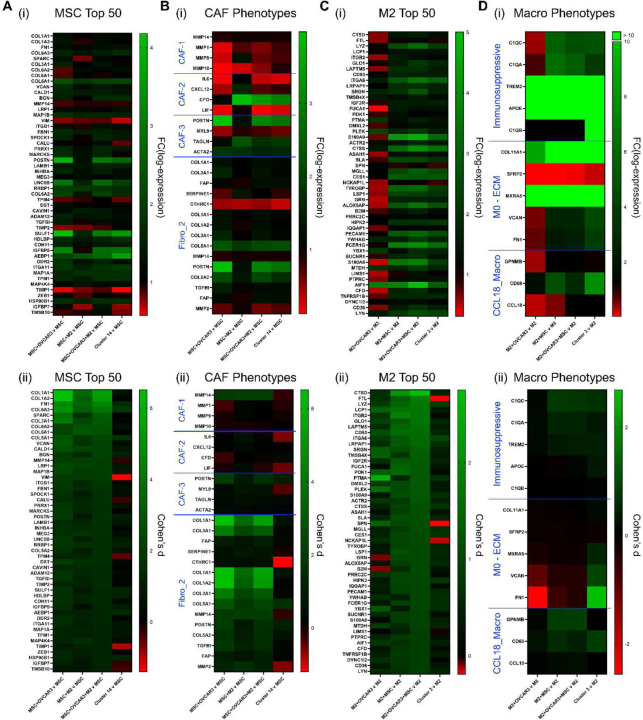
MSCs adopt myofibroblastic phenotype and macrophages adopt immunosuppressive and ECM-associated phenotype in triculture. **A)** The 50 genes in MSC with the largest effect size between tri-component tumoroids and MSC mono-spheroids were identified. (i) Fold change in gene log-expression and (ii) Cohen’s d effect size are displayed. **B)** Fibroblast gene signatures were evaluated in MSC from the tri-component and co-component tumoroids, and an increase in gene expression associated with myofibroblasts and other tumor associated fibroblasts were observed in MSC from OVCAR3+MSC, OVCAR3+MSC+M2, and ***Cluster 14***. **C)** The 50 genes in M2 with the highest fold-change between tri-component tumoroids and M2 mono-spheroids were identified. **D)** Macrophage gene signatures were evaluated in M2 from the tri-component and co-component tumoroids, and an increase in gene expression associated with immunosuppressive and ECM-associated macrophage phenotypes were observed in M2 from MSC+M2, OVCAR3+MSC+M2, and ***Cluster 3***.

The stromal cells of the tumor microenvironment play an important role in influencing the phenotype of HGSC cells. CA-MSC, CAF, and M2-AAM have been shown to support phenotypes such as chemoresistance, stemness, migration, and metastasis which are associated with worse outcomes for HGSC patients.^[3,4,6,7,22–28]^ Therefore, in addition to investigating the changes observed in HGSC cells within the tumoroids, we also investigated the changes in stromal cells due to the presence of the other cells in the tumoroids. Expression of genes associated with different CAF and macrophage phenotypes were evaluated from scRNA-seq of tri-component tumoroids and spheroids. Myofibroblastic CAF markers *POSTN, ACTA2, and TAGLN* were found to be the most enriched in co- and tri-tumoroids. *ACTA2* and *TAGLN* encode cytoskeletal proteins that play an important role in fibroblast cell motility and contraction.^[74,75]^ *POSTN* encodes an extracellular matrix protein associated with cell migration and has been shown to recruit M2 macrophages in ovarian cancer.^[76,77]^ Increased *POSTN* was observed with fold change log-expression values of 1.23, 3.98, 3.02, and 2.74 and Cohen’s d values of 0.06, 1.36, 1.03, and 1.01 for MSC+M2, OVCAR3+MSC, OVCAR3+MSC+M2, and ***Cluster 14*** compared to MSC-only spheroids, respectively (Fig. 7A). *ACTA2* was increased with fold change log-expression values of 1.76, 1.83, 1.50, and 1.58 and Cohen’s d values of 0.12, 0.12, 0.11, and 0.13 for MSC+M2, OVCAR3+MSC, OVCAR3+MSC+M2, and ***Cluster 14*** compared to MSC-only spheroids, respectively. Additionally, *TAGLN* was increased with fold change in log-expression of 2.25, 1.13, 1.68, and 1.84 and Cohen’s d values of 0.27, 0.01, 0.23, and 0.25 for MSC+M2, OVCAR3+MSC, OVCAR3+MSC+M2, and ***Cluster 14*** compared to MSC-only spheroids, respectively.

The macrophages in co- and tri-component tumoroids were found to express markers related to the M0-ECM phenotype identified by Puttock, et al. and the immunosuppressive phenotype reported by Zhang, et al.^[78,79]^ *FN1* was increased in all but OVCAR3+M2 with fold change in log-expression of 0.62, 2.15, 2.38, and 3.87 and Cohen’s d values of −0.49, 1.09, 1.82, and 2.69 in OVCAR3+M2, MSC+M2, OVCAR3+MSC+M2, and ***Cluster 3*** compared to M2-only spheroids, respectively (Fig. 7B). *VCAN* was similar, with fold change in log-expression of 0.55, 2.69, 2.77, and 4.47 and Cohen’s d values of −0.09, 0.53, 0.78, and 0.94 in OVCAR3+M2, MSC+M2, OVCAR3+MSC+M2, and ***Cluster 3*** compared to M2-only spheroids, respectively. *C1QC* showed fold change log-expression of 0.42, 5.50, 4.59, and 4.82 and Cohen’s d of −0.19, 0.67, 0.62, and 0.68 for OVCAR3+M2, MSC+M2, OVCAR3+MSC+M2, and ***Cluster 3*** compared to M2-only spheroids, respectively. *Q1QA* showed fold change log-expression of 0.72, 6.07, 5.31, and 3.59 and Cohen’s d of 0.11, 0.52, 0.49, and 0.32 for OVCAR3+M2, MSC+M2, OVCAR3+MSC+M2, and ***Cluster 3*** compared to M2-only spheroids, respectively (Fig. 7B). Overall, these gene expression profiles demonstrate that the presence of HGSC cells and other stromal cells (MSC or M2) in the tumoroids influence MSC to adopt a more myofibroblastic phenotype, and influence M2 to adopt a more ECM-associated and immunosuppressive phenotype, which are all associated with worse outcomes in HGSC.

### EMT cluster in tumoroids.

**Figure 8:**
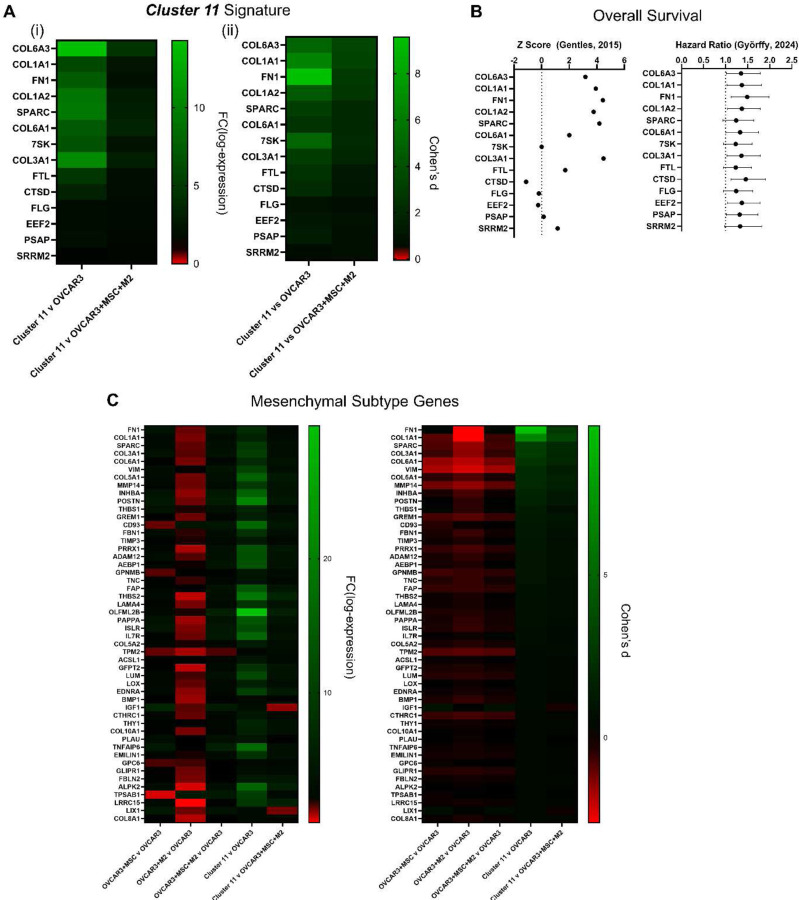
Unique Cluster 11 in tri-component tumoroids associated with EMT, mesenchymal molecular subtype, and worse overall survival in patients. **A)** The ***Cluster 11*** gene signature (*COL6A3, COL1A1, FN1, COL1A2, SPARC, COL6A1, 7SK, COL3A1, FTL, CTSD, FLG, EEF2, PSAP, SRRM2*) was generated by finding the overlapping genes with the highest expression and highest AUC values. (i) Fold change in gene log-expression and (ii) Cohen’s d effect size are displayed. **B)** The genes from the ***Cluster 11*** signature were correlated with shorter overall survival using the PRECOG database and Kaplan-Meier Plotter.^[80–82]^ **C)** The top 50 genes in the mesenchymal subtype were compared across the co- and tri-component tumoroid conditions, and the highest expression of these genes was observed in ***Cluster 11***.^[8]^

In the scRNA-seq, the unique ***Cluster 11*** was located in between the OVCAR3 and MSC clusters on the UMAP plot and its position between the epithelial cancer cells and mesenchymal stem cells suggested shared features of each population. Additionally, the EMT pathway was upregulated for ***Cluster 11*** in GSEA compared to OVCAR3 cells from all tumoroid conditions. Therefore, this cluster was suspected to be cancer cells undergoing EMT and was further investigated. To establish a gene signature for ***Cluster 11***, the 50 genes with the highest expression values (Supplementary Figure S7A) and the genes with the 50 highest AUC values in the ***Cluster 11*** vs OVCAR3 tri-component comparison (Supplementary Figure S7B) were compared and the overlapping genes were selected to represent ***Cluster 11***. This resulted in the 14 gene ***Cluster 11*** signature: *COL6A3*, *COL1A1*, *FN1*, *COL1A2*, *SPARC*, *COL6A1, 7SK*, *COL3A1*, *FTL*, *CTSD*, *FLG*, *EEF2*, *PSAP, SRRM2* (Fig. 8A). These genes were input into the PRECOG database (https://precog.stanford.edu/) and Kaplan-Meier Plotter to evaluate their correlation with overall survival.^[80–82]^ Most of the genes in ***Cluster 11*** were associated with shorter overall survival; however, *7SK*, *CTSD* and *FLG* had mixed results, with PRECOG reporting a lower z score and Kaplan-Meier Plotter reporting a hazard ratio (HR) greater than 1. *COL3A1*, *FN1*, and *SPARC* had the strongest association with decreased overall survival, with meta-Z scores of 4.49, 4.44, and 4.19 and HR of 1.37, 1.49, and 1.24, respectively (Fig. 8B).

Many of the genes in the ***Cluster 11*** signature are ECM proteins and are associated with the C1 mesenchymal molecular subtype of HGSC, which is also associated with worse prognosis in HGSC patients.^[8]^ Therefore, the top 50 genes identified in the Tothill et al. mesenchymal subtype were examined in our tumoroid conditions and ***Cluster 11***. The expression of these mesenchymal genes were enriched in ***Cluster 11*** compared to OVCAR3 from mono-spheroids or tri-component tumoroids (Fig. 8C). Overall, the gene expression of this unique ***Cluster 11*** indicates that the tri-component tumoroids support an EMT phenotype in the HGSC cells and recreates the mesenchymal molecular subtype signature of HGSC. However, others have suggested that the mesenchymal signature in tumors can be attributed to the stromal cells within the tumor, since these sequencing analyses for subtype classification use bulk tumor samples, not specifically cancer cells.^[5,83]^ Our results indicate that the stromal MSC and M2 express genes from the mesenchymal subtype signature as well (Supplementary Figure S8). However, we have identified a subset of cancer cells, ***Cluster 11***, which also contribute to this signature, indicating that it is not strictly the stromal cells, but also some of the cancer cells. The ***Cluster 11*** cells comprise approximately 13.6% of the OVCAR3 cells in the tri-component tumoroids (Supplementary Table S2). Therefore, our results suggest that there is a subset of cancer cells which acquire this mesenchymal subtype signature, potentially via epithelial to mesenchymal transition. This subset of cells could be a promising target for new therapeutic agents.

## Discussion

The tumor microenvironment of ovarian cancer is rich in macrophages, CA-MSC, and CAF.^[3–7]^ These cell types can enrich cancer stem-like cell (CSC) populations and promote chemoresistance and metastasis.^[3–5,7,19–28]^ However, *in vitro* 3D models to systematically study the multi-directional relationship between cancer cells, macrophages, and CA-MSC, are lacking for HGSC. In breast and pancreatic cancers, CA-MSC and CAF have been observed to have crosstalk with macrophages via CSF-1 secretion which enhances an immunosuppressive stroma phenotype.^[84,85]^ Gastric cancer-derived MSC have been shown to prime macrophages toward the M2 phenotype, and cancer cells exposed to these gastric cancer-MSC primed macrophages showed enhanced migration and invasion potential.^[86]^ Meanwhile, macrophages primed with CXCL12 overexpressing MSC resulted in enhanced breast tumor induction in mice.^[87]^ Exosomes from tumor-educated MSC induced monocytic myeloid derived suppressor cells into M2 macrophages in a breast cancer model, which accelerated tumor growth.^[88]^

Given the abundance of macrophages and CA-MSC in the tumor microenvironment of HGSC and these previously observed MSC-macrophage interactions in other cancer types, we implemented a 3D tri-component tumoroid platform incorporating HGSC cancer cells, MSC, and macrophages to study the multi-directional signaling between these three cell types and their impact on HGSC cell phenotypes. This platform brings each cell type into close contact with each other in the setting of a 3D self-assembling microtissue and a fluidic environment which reproduces key features of the tumor and malignant ascites in patients.^[56,57,89–91]^ In order to distinguish each cell type independently, we transduced each cell type to express a fluorescent protein and detected them using FACS. Each cell type remained viable in single-, co-, and tri-component spheroids and tumoroids respectively.

Resistance to chemotherapy is a major challenge in treatment of HGSC, with 70% of patients developing recurrent tumors. Therefore, the response to first line chemotherapeutic agents, carboplatin and paclitaxel, was evaluated in the OVCAR3, OVCAR4, and OVCAR8 tumoroids which showed varying responses to these agents. The tumoroids and spheroids tolerated higher concentrations of these agents compared to IC50 values reported in the literature (50–80 µM IC50 for carboplatin, 5–18 nM IC50 for paclitaxel), which were determined using cells in 2D culture.^[39]^ This is consistent with previous findings showing HGSC cell lines in 3D are more resistant to chemotherapy.^[57,91,92]^ Additionally, literature showed OVCAR8 was more sensitive to carboplatin and paclitaxel, and OVCAR3 was most resistant to carboplatin, which is consistent with the response of the tumoroids and spheroids in this study.^[39]^ Furthermore, the addition of MSC and/or M2 to the HGSC tumoroids resulted in increased resistance to these agents. This is supported by previous studies that identified CA-MSC and macrophages as promoters of chemoresistance in HSGC.^[4,6,7,93]^ Interestingly, the addition of both MSC and M2 into HGSC tumoroids did not appear to have a synergistic effect over the addition of only MSC or only M2. This could be due to already high levels of drug resistance or overlapping mechanisms of resistance in the tri-component tumoroids.

The expression of cancer stem-like cell markers was investigated in the tumoroids since chemoresistance is often attributed to the presence of cancer stem-like cells.^[94–98]^ Increases in stem markers CD44, CD24, and CD90 were observed with the addition of MSC and macrophages to the tri-component tumoroids. ALDH activity at baseline was higher than reported in literature for OVCAR3 and OVCAR4, which have reported levels of ALDH activity of 3.2–26.5% of cells and 4.1% of cells, respectively, whereas OVCAR8 tumoroids remained with low levels of ALDH, more consistent with literature values of 0.49–1.9% of cells.^[54,99–101]^ The higher ALDH activity in our tri-component tumoroid platform is likely due to the tumoroids enriching for cancer stem-like cells in the attachment-free suspended fluidic environment. This effect is likely enhanced in OVCAR3 and OVCAR4 since these cell lines are reported to have higher proportions of cells with ALDH activity at baseline than OVCAR8. These observations are consistent with findings showing MSC and macrophages inducing more stem-like phenotypes in ovarian cancer, as well as other cancer types.^[3,4,6,7,25–28,102,103]^ There did not appear to be a synergistic increase in stem marker expression when MSC and M2 were both present in tri-component tumoroids compared to co- or mono-spheroids. This pattern is consistent with the level of chemotherapy response observed in the tumoroids, which did not demonstrate a synergistic response with both MSC and M2 present. Additionally, MSC-comprising tumoroids often featured both higher resistance to chemotherapy and higher stem marker expression, indicating that MSC are more influential in producing these phenotypes than M2 in this 3D tri-component tumoroid platform. Furthermore, given that the FDA has recently approved the anti-CD44 antibody drug conjugate (ADC) treatment for HGSC, the increased CD44 expression observed in the tumoroids reinforces the utility of this targeted therapy and suggests that the anti-CD44 antibody could reduce the presence of cancer stem-like cells promoted by MSC and macrophages in the ovarian tumor microenvironments.^[60]^

Metastasis is a major challenge in the treatment of ovarian cancer, with the majority of patients diagnosed at advanced stages where tumors have already spread throughout the peritoneal cavity.^[61]^ To assess metastatic potential *in vitro*, we utilized migration and invasion assays. More HGSC cells migrated through a transwell membrane in response to conditioned medium from tumoroid with MSC and M2 added, compared to HGSC-only spheroids or fresh medium. This indicates that soluble signals from MSC and M2 increase migration in cancer cells. Interestingly in the assay of invasion of a mesothelial cell monolayer, there was significantly more invasion when spheroids contained MSC compared to M2. This suggests that the presence of the MSC, and not simply their soluble signals, is critical for invasion of HGSC tumoroids through a mesothelial cell layer.

In order to determine the signaling between MSC, macrophages, and HGSC that drive these observed phenotypes, scRNA-seq was performed on the tumoroids. During cell clustering, a unique cluster was identified that did not cluster with either of the three cell types. This unique cluster (***Cluster 11***) was found in the OVCAR3+MSC+M2 tri-component tumoroids only. This cluster contained mostly GFP and EpCAM expressing cells, but also contained cells expressing smURFP and CD73. Therefore, this cluster was analyzed separately from the other cell types. GSEA hallmark analysis revealed that epithelial to mesenchymal transition (EMT) was activated in OVCAR3 from OVCAR3+MSC+M2 tumoroids compared to OVCAR3 spheroids. Furthermore, EMT was further activated in ***Cluster 11*** compared to OVCAR3 from OVCAR3+MSC+M2 tumoroids and OVCAR3 spheroids. This is consistent with the increased migration and invasion observed in the tri-component tumoroids. The increase in EMT is also consistent with previous studies showing increased EMT of ovarian cancer cells in the presence of MSC or macrophages separately.^[7,104–106]^ EMT has also been associated with lower disease-free survival in ovarian cancer.^[107]^ Additionally, WNT/β-catenin signaling was activated in ***Cluster 11*** compared to OVCAR3 from OVCAR3+MSC+M2 tumoroids and OVCAR3 spheroids. WNT is one of the signaling pathways involved in inducing EMT, which aligns with increased EMT in the ***Cluster 11***.^[108]^ Specifically, WNT5A and WNT5B were the dominant Wnt ligands produced by MSC and signaling to OVCAR3. WNT5A has been identified as the highest expressed Wnt ligand in CAFs which enriches stem-like features in neighboring ovarian cancer cells and supports chemoresistance via noncanonical Wnt signaling.^[109–115]^ WNT5B has not been identified in CA-MSC or CAF crosstalk with ovarian cancer cells; however, it has been observed to be secreted by macrophages to promote more stem-like, chemoresistance, and invasive ovarian cancer cells.^[6,116,117]^ TGF-β signaling was also increased in ***Cluster 11*** and ligand-receptor analysis revealed that both MSC and macrophages activate TGF-β pathway receptors in ovarian cancer cells. Overall, the increase in EMT and WNT signaling observed in metastatic HGSC are concordant with their activation in the tri-component tumoroids. Therefore, the tumoroid platform reproduces the cell-cell interactions in the metastatic HGSC tumor microenvironments.

The gene expression of MSC and macrophages in the co- or tri-component tumoroids was also analyzed to determine how they responded to the presence of the other stromal cells and which of their signaling pathways were most activated. MSC from OVCAR3+MSC+M2 tri-component tumoroids, OVCAR3+MSC co-tumoroids, MSC+M2 spheroids, and ***Cluster 14*** had increased expression of myofibroblastic CAF markers compared to MSC-only spheroids. This is consistent with the ability of MSC to differentiate into CAF in the tumor microenvironment.^[118,119]^ The ability of the tri-component tumoroid platform to induce and support a myofibroblastic CAF phenotype therefore enables it to be ideally suited for the study of cell-cell interactions and screen drugs, since tumors with myofibroblastic CAFs have poorer prognosis than tumors with few CAFs.^[5,79,120–122]^ The MSC in the tri-component tumoroids upregulated many genes found in the Fibro_2 CAF subtype from Deng et al.^[123]^ These Fibro_2 CAFs were found to contribute to EMT, and the tumoroids in which these MSC with Fibro_2-like signatures are found, also have activation of the hallmark EMT pathway. Furthermore, the gene expression profile of macrophages in the tri-component tumoroids revealed an immunosuppressive and ECM-associated phenotype. This immunosuppressive phenotype was previously identified in chemoresistant metastatic ovarian cancer and can provide protection from other immune cells.^[79]^ The macrophages from tri-component tumoroids also upregulated genes found in the CCL18_Macro population which are involved in recruitment of immunosuppressive myeloid cells.^[123]^ ***Cluster 3*** macrophages found in the tri-component tumoroids expressed genes upregulated in the ECM-associated macrophage phenotype, which is associated with poor prognosis and impaired anti-cancer immune activity, which is consistent with the immunosuppressive phenotype also observed in these cells.^[78]^ The ability to reproduce these clinically relevant phenotypes of stromal cells (both CAF and macrophage) within the HGSC tumor microenvironment is a valuable feature of our tri-component tumoroid platform.

Finally, the gene expression profile of the unique ***Cluster 11*** with EMT features was used to create a signature for this population and compared against the metastatic tumors in TCGA dataset. The ***Cluster 11*** gene signature is associated with lower overall survival in ovarian cancer patients. Additionally, many of the genes in this signature are associated with more aggressive phenotypes in other solid tumors. For instance, *COL6A3, COL1A1, COL1A2, COL6A1, COL3A1* and *FN1* were among the most upregulated in ***Cluster 11*** and overexpression of collagens is often seen in aggressive solid tumors including in pancreatic cancer and triple negative breast cancer.^[124,125]^ *COL6A3, COL3A1, COL1A2*, and *FN1* are overexpressed in pancreatic cancer and associated with worse prognosis, malignant progression, and metastasis.^[124,126–130]^ *COL6A1* is also associated with metastasis and worse outcomes in pancreatic cancer and *COL1A1* promotes migration and EMT in pancreatic cancer.^[131,132]^ Similarly, *COL1A1, COL3A1*, and *FN1* are overexpressed in breast cancer and contribute to proliferation and invasion.^[125]^ *FN1* has similar effects, but also contributes to chemoresistance and is associated with poor prognosis in breast cancer and overexpressed in triple negative breast cancer.^[133–135]^ *COL6A1 and COL6A3* are also associated with lower overall survival in breast cancer and *COL6A3* expression can predict EMT and metastasis in triple negative breast cancer.^[125,136]^ Taken together, the tri-component tumoroids enabled features associated with more aggressive HGSC to be recapitulated in an *in vitro* 3D model. Our highly controllable tri-component tumoroid platform can be used to investigate cell-cell interactions, particularly in the mesenchymal molecular subtype of HGSC, and identify new drug targets for treatment of these tumors that have EMT phenotypes and poorer outcomes.

## Conclusion

The microenvironments of high-grade serous ovarian cancer (HGSC) metastatic tumors are diverse, containing multiple cell types that drive tumor progression. Despite the critical importance of these interactions, analyzing the underlying mechanisms remains challenging due to the complexity of *in vivo* models and the limitations of standard *in vitro* culture systems. In order to study these multi-directional interactions, we developed a non-adherent tri-component tumoroid platform that incorporates HGSC cells, macrophages, and carcinoma-associated mesenchymal stem cells. Using this biologically-relevant and high-fidelity 3D suspended tumoroid platform, we sought to identify their multi-directional interactions responsible for the metastatic and chemoresistant HGSC. Our tri-component tumoroid platform promoted cell phenotypes relevant to clinically aggressive tumors which have been challenging to treat and have poor prognosis. These features captured by the tumoroid platform include resistance to chemotherapeutic agents carboplatin and paclitaxel, increased expression of cancer stem-like markers, increased migration and invasion, and activation of EMT, Wnt/β-catenin, and PI3K/Akt/mTOR pathways. This platform can be used to understand the cell-cell interactions between these three cell types to uncover novel therapeutic strategies and can be used as a high-throughput drug screening platform. The fine control over the number of each cell type present in each tumoroid provides a highly reproducible model which is amenable to characterization of the contribution of each non-tumor stromal cell type. We believe that the tri-component tumoroids can be leveraged in translational research, bioengineering, and cancer immunology to discover and characterize novel targets for HGSC and other solid cancers.

## Materials and Methods

### Materials.

Ovarian cancer cell lines OVCAR3, OVCAR4, and OVCAR8 were obtained from the National Cancer Institute (NCI) Division of Cancer Treatment and Diagnosis (DCTD) Tumor Repository. Monocyte cell line U937 was purchased from ATCC (Manassas, VA). Human adipose derived MSC were purchased from Lonza Bioscience (Lonza Walkersville Inc., MD). Mesothelial cell line LP-9 was obtained from the Corielle Institute for Medical Research (Camden, NJ). Cells were confirmed negative of mycoplasma contamination using the PlasmoTest mycoplasma detection kit (InvivoGen, San Diego, CA). Cell line purity was confirmed with STR profiling (Labcorp, Inc., Burlington, NC). Tissue culture reagents were purchased from Life Technologies, cytokines were purchased from Peprotech Inc (Thermo Fisher Scientific), and chemicals were purchased from Sigma Aldrich (St Louis, MO) unless otherwise noted. Lentiviral vectors were purchased from Sigma Aldrich and Addgene (Watertown, MA) and packaged at the University of Michigan Viral Vector core.

### Differentiation and polarization of macrophages from U937.

Macrophages were generated from U937 as described previously.^[6]^ Briefly, U937 were maintained in suspension culture in RPMI 1640 supplemented with 10% heat-inactivated fetal bovine serum and 1x antibiotics/antimycotics. U937 were differentiated into M2-like alternately activated macrophages (M2-AAM, henceforth called ‘M2’ in this report) by plating them in hanging drops at a density of 3,000 cells/well with 5 ng/mL PMA. After 24 hours, IL4 and MCSF were added to the cells to a final concentration of 20 ng/mL for 48 hours. After differentiation and polarization, U937-derived macrophages demonstrate significantly higher CD163 expression than untreated controls via flow cytometry (Supplementary Figure S9). Spheroids were collected, dissociated, and counted for use in experiments.

### Formation of spheroids and tumoroids.

HGSC cell lines OVCAR3, OVCAR4, and OVCAR8 were maintained in RPMI 1640 supplemented with 10% FBS and 1x antibiotics/antimycotics. Mesenchymal stem cells were maintained in AD-SCBM (Lonza) supplemented with 10% FBS, 1x antibiotics/antimycotics, and 1% L-glutamine. Adherent cells were collected using trypsin and counted. Macrophages were differentiated and counted as indicated above. Cells were resuspended in complete RPMI 1640 at a concentration of 30,000 cells/mL or 600 cells/well for each cell type included in the sample. Cell suspensions were plated in hanging drop arrays with 20 µL/drop to form spheroids, as described previously.^[91,137,138]^ Spheroids were imaged and fed with 2 µL complete RPMI on day 3. Spheroids were imaged and collected for analysis on day 5. To dissociate spheroids into single cell suspensions, spheroids were collected from hanging drops using a pipette and resuspended in 0.25% trypsin for 7 minutes at 37 °C.

### Characterization of three cell types in spheroids.

OVCAR3, OVCAR4, and OVCAR8 were transduced with a lentiviral vector pLenti-EV-GFP-VSVG to express GFP. MSC were transduced with a lentiviral vector pLenti-smURFP to express smURFP. U937 were transduced with a lentiviral vector pLVX-IRES-mCherry-VSVG to express mCherry. After transduction, cells were sorted using FACS to collect the cells positive for their respective fluorescent protein. Spheroids were generated with these fluorescently labeled cells. Confocal microscopy was performed on live spheroids in hanging drops using a Nikon A1R HD. Spheroids were also dissociated into single cell suspensions and resuspended in PBS supplemented with 2% FBS and 1 µg/mL DAPI for flow cytometric analysis on ThermoFisher Bigfoot at the University of Michigan Flow Cytometry Core.

### Chemoresistance of spheroids.

Spheroids were imaged 72 hours after plating the cells. After imaging, the spheroids were treated with carboplatin (LKT Laboratories, St Paul, MN), paclitaxel (APExBIO, Houston, TX), or both. 2 µL of drug solution was added to each spheroid to achieve a final concentration of 10 µM paclitaxel or 300 µM carboplatin. Vehicle controls were generated by treating spheroids with equivalent volumes of solvent (ultrapure DI water for carboplatin and DMSO for paclitaxel). The spheroids were incubated with the drug for 48 hours and then collected for flow cytometry analysis. Spheroids were dissociated into single cell suspensions and resuspended in DAPI buffer. Data was collected using an Attune NxT flow cytometer. The proportion of viable OVCAR3 and OVCAR4 cells was determined by gating on the GFP-labeled cancer cells and then recording the proportion that were viable as indicated by cells negative for DAPI stain. The viability was normalized to the drug-free control sample for each spheroid condition. The proportion of viable OVCAR8 cells was determined by analyzing an equal volume of sample for each condition and recording the total number of GFP-labeled cancer cells negative for DAPI stain. The live OVCAR8 cell numbers were normalized to the drug-free control sample for each spheroid condition.

### Flow cytometry for cancer stem-like markers in spheroids.

Spheroids were collected on day 5 and dissociated into single cell suspensions. Cells were resuspended in staining buffer and divided into separate tubes for each antibody. Antibodies against cancer stem-like cell markers were added: CD44-APC (BD Pharmingen, Franklin Lakes, NJ), CD90-PE (eBioscience, Thermo Fisher Scientific), CD133-APC (BD Pharmingen), CD24-APC (Biolegend, Inc, San Deigo, CA) as well as their respective isotype controls: IgG2b, κ-APC (BD Pharmingen), IgG1, κ-PE (Biolegend, Inc.), IgG1, κ-APC (BD Pharmingen), IgG2a, κ-APC (Biolegend, Inc.). Cells were incubated with antibodies at 37 °C for 30 minutes. The antibody was then removed, and cells were resuspended in DAPI buffer and kept on ice for flow cytometric analysis on Attune NxT. To assess ALDH activity, the AldeRed (Sigma Aldrich) was used. Cells were resuspended in AldeRed Assay Buffer and AldeRed reagent was added. A paired tube also received DEAB reagent to inhibit ALDH activity and serve as a background control. Cells were incubated at 37 °C for 30 minutes. Excess reagent was removed and cells were resuspended in DAPI buffer and kept on ice for flow cytometric analysis on Attune NxT. Using FlowJo analysis software, gates were set by first filtering out doublets and dead cells, then selecting only GFP+ HGSC cells, and setting the background to 0.5% nonspecific binding using the isotype control. The resulting proportion of positively labeled cancer cells was recorded for each marker and spheroid condition.

### Conditioned medium transwell for migration of spheroids.

600 µL conditioned medium collected from spheroids or tumoroids on day 5 was placed in the bottom chamber of a transwell plate (Corning). 100,000 HGSC cells were added to the top of the transwell membrane in 200 µL of starved RPMI 1640. The same HGSC cell line that was used to generate the spheroid or tumoroid was placed on top of the transwell membrane. Cells migrated for 24 hours, then membranes were fixed with 10% formalin for 10 minutes, permeabilized with methanol for 30 minutes, and stained with 0.5% crystal violet for 20 minutes. Cells were removed from the top of the membrane using a cotton swab. The migrated cells were left on the bottom of the membrane and imaged using an Olympus IX83 inverted microscope equipped with U-HGLGPS fluorescence light source and Hamamatsu camera controller. Five non-overlapping images were taken per membrane and the area covered by migrated cells was quantified using ImageJ.

### Invasion of spheroids through LP-9 monolayer.

Spheroids or tumoroids made with GFP-labeled HGSC cells were collected on day 5 and plated on top of an mCherry-labeled LP-9 monolayer in 96 well plates. Spheroids or tumoroids were allowed to attach for 2 hours, then fluorescent images were taken of the LP-9 monolayer and spheroids. Images were acquired of each spheroid or tumoroid at 2 hours, 24 hours, 48 hours, and 120 hours after addition to the LP-9 monolayer. The area of the spheroid or tumoroid was quantified using ImageJ. The size of the void in the LP-9 monolayer was quantified by outlining the void area and then quantifying the area with ImageJ. The void area was normalized to the initial area of the spheroid or tumoroid.

### Sample preparation for single-cell RNA sequencing of tumoroids.

Spheroids were generated with 600 cells/well of each cell type. On day 5, spheroids were collected, dissociated into single cells, and fixed. Cells were then prepared using the Parse Single Cell Whole Transcriptome kit and sequenced at the University of Michigan Advanced Genomics Core.

### Single-cell RNA sequencing analysis.

Unless otherwise stated, all tools, software, and functions mentioned throughout this section were executed using their default parameters as described in their respective documentation. Specific adjustments and non-default parameters have been explicitly detailed where applicable.

### Preprocessing: single-cell RNA sequencing.

The barcode whitelist was generated by utilizing a combinatorial approach based on the barcoding schema of Parse Biosciences. The barcode data were obtained from the sequencing run and included barcodes from three rounds of barcoding (Rounds 1, 2, and 3) along with their associated metadata, such as type (T or R) and well positions. First, barcodes were grouped by their respective barcoding rounds and categorized into T-type and R-type for Round 1. For each barcode type, a comprehensive set of barcode combinations was created by iteratively concatenating the barcode sequences from Round 3, Round 2, and Round 1. Each combination represented a unique barcode for indexing reads back to their original well or template. The well information for Round 1 barcodes was also propagated to the generated barcodes to maintain experimental context. The final whitelist consisted of two barcode sets: one for T-type and one for R-type, ensuring all possible combinations were captured. The resulting whitelist was stored as a structured file for downstream demultiplexing and read assignment.

### Gene-count: single-cell RNA sequencing.

Gene-level read counts were obtained using the *Kb-python* pipeline^[139]^ which utilizes pseudo-alignment via *kallisto*^[139]^ to assign reads to the transcripts. The reference index included annotated transcripts from the genome along with ERCC spike-in sequences. Barcodes were demultiplexed using *bustools*^[139]^, guided by the whitelist of valid barcodes. To address potential barcode inconsistencies, a custom R script was employed to collapse barcodes. This script matched observed barcodes to entries in the whitelist, aggregated gene counts for each unique barcode, and exported the collapsed counts in the equivalence class matrix format. The equivalence matrix file was loaded into R in the form of dgCMatrix using the *read_count_output* function in the *BUSpaRse*^[139]^ package. The cells were then segregated into their respective sample (OVCAR3+MSC+M2, OVCAR3+MSC, OVCAR3+M2, MSC+M2, OVCAR3, MSC and M2) SingleCellExperiment (SCE) objects by a custom R function *separate_samples.* Mitochondrial genes and ERCC were omitted from the matrices during the mentioned conversion into SCE objects.

### Initial filtering: single-cell RNA sequencing.

The *emptyDrops* function, from the Bioconductor package *DropletUtils*^[140]^ was used to identify droplets containing ambient RNA, with statistical significance assessed using 20,000 iterations and parallel processing (*MulticoreParam* (20)) for each sample SCE object separately. Droplets classified as empty were removed. Subsequently, quality control metrics were applied using the *perCellQCFilters* function from *Scuttle*^[141]^ to flag cells based on mitochondrial RNA content, library-size and detected features. Cells failing these thresholds were excluded.

### Doublet detection: single-cell RNA sequencing.

For each sample, pre-clustering was performed using *quickCluster* from *scran*^[142]^ to group cells with similar expression profiles. Scaling factors were then calculated using *computeSumFactors* (min.mean = 0.1), which leverages these pre-clusters to account for cell population heterogeneity during normalization. These scaling factors were subsequently used to compute log-normalized counts for each sample independently using *logNormCounts* from *Scuttle.*^[141]^ Doublets were identified for individual samples using *scDblFinder.*^[143]^ Clustering information guided doublet detection, with *scDblFinder* predicting doublets based on cell-to-cell similarity.

### Dimensionality reduction: single-cell RNA sequencing.

To enable combined visualization of all seven scRNA-seq samples, the datasets were integrated into a single SingleCellExperiment object. Within-batch normalization was first performed using *multiBatchNorm* from *batchelor*^[144]^ to adjust for differences in library sizes across batches. Highly variable genes (HVGs) were identified within each batch using *modelGeneVar* and the variability statistics were aggregated across batches using *combineVar* from *scran.*^[142]^ From this combined variability profile, the top 5,000 HVGs were selected for integration using *getTopHVGs.*^[142]^

The normalized samples were then combined into a unified dataset using *correctExperiments*^[144]^ with *NoCorrectParam*, which concatenates batches without applying explicit batch effect correction. Principal Component Analysis (PCA) was performed on the combined dataset, using *runPCA*,^[142]^ leveraging the selected HVGs for dimensionality reduction, and the proportion of variance explained by each principal component was evaluated. Uniform Manifold Approximation and Projection (UMAP) was subsequently applied to the PCA-transformed data, using *runUMAP*,^[142]^ to visualize the integrated dataset, with cells colored by their original sample source to assess the presence of batch-specific biases.

PCA was performed using the selected HVG *logCounts*, and the explained variance was plotted to determine the optimal number of principal components for subsequent steps. The elbow point of the variance plot was used to guide dimensionality selection. UMAP was applied to the PCA-transformed dataset using a minimum distance of 0.6 and 250 neighbors for visualization. Cells were colored by sample to examine clustering patterns and relationships.

### Clustering: single-cell RNA sequencing.

To refine the integrated dataset for downstream analysis, cells identified as doublets and with library sizes less than or equal to 2^^10^ were filtered out. HVGs were identified using *modelGeneVar*,^[142]^ and the top 500 HVGs were selected with *getTopHVGs*^[142]^ to focus dimensionality reduction on biologically relevant features.

For clustering, a two-step strategy was implemented using the *TwoStepParam* function from *bluster.*^[145]^ The first step involved k-means clustering with 1,000 centers, followed by graph-based clustering with k=5. The resulting cluster labels were assigned to cells and saved for downstream interpretation. The processed dataset, including PCA and UMAP embeddings, was saved for further analyses.

### Cluster annotation: single-cell RNA sequencing.

Fluorescent marker expression (GFP, mCherry, and smURFP) and cell-type markers (e.g., THY1, PTPRC, EPCAM) expressions (normalized log-transformed counts) were overlaid on the UMAP to distinguish cell types and validate cluster assignments. Cells expressing mesenchymal stem cell (MSC) markers (CD73, CD90, CD105), immune markers (CD4, CD45), and epithelial markers (EPCAM) were analyzed using density plots generated with *Nebulosa.*^[146]^ Expression thresholds were defined as log-transformed counts greater than 0.15 for cell-type markers. Similarly, fluorescent marker expression was assessed using a threshold of log-transformed counts greater than 1.

Dual- and triple-fluorescent marker expressing cells were flagged based on co-expression of multiple markers (e.g., CD73 and EPCAM, CD45 and EPCAM) and excluded from subsequent analyses to minimize bias. Cluster-specific distributions of mitochondrial content, library size, and detected features were also visualized to ensure data quality.

Marker expression differences across clusters were evaluated to assign putative cell identities. Subset analyses focused on key clusters of interest (e.g., clusters 3, 11, and 14) to explore intra-cluster variability and identify potential subpopulations. UMAP projections were regenerated, and clusters were further subdivided using additional graph-based clustering. Specific marker genes (e.g., *FLG* and *MUC16*) were analyzed in these clusters to refine cell-type identification.

This multi-layered approach, leveraging *Nebulosa*^[146]^ for density plots and systematic thresholds for expression calling, provided robust identification of cell populations and resolved ambiguities in cluster assignments, supporting downstream biological interpretations.

To provide additional confidence in the marker-based annotation calls, *SingleR*^[147]^ was independently employed for automated cell annotation. *SingleR* mapped the single-cell transcriptomes against the *BlueprintEncodeData* from *celldex*^[147]^ reference dataset, which comprises known transcriptomic profiles for well-defined cell types. Predictions were generated using the *SingleR* function, where the best matching reference labels were assigned to each cell based on similarity. Parallel computations were executed across 20 cores using *BiocParallel::MulticoreParam* for efficiency. The annotation confidence was evaluated by visualizing prediction scores and cell-type assignments in heatmaps generated with *pheatmap*, which incorporated log-transformed counts with a pseudo-count of 10 to ensure smooth visual representation.

Cluster 3, 11 and 14 were further explored for subpopulations and marker-specific expression using *scater*. Highly variable genes were identified using *modelGeneVar*, and the top 10,000 features were selected for dimensionality reduction. Principal component analysis (PCA) was performed with six components, and the explained variance was inspected to determine the optimal number of dimensions for downstream analysis. UMAP was applied using *runUMAP*, with parameters set to a minimum distance of 0.1 and 5 neighbors, focusing on 2,000 top features. Marker expressions (e.g., GFP, mCherry, smURFP, and key cell-type markers such as EPCAM, CD45, and CD90) were visualized on PCA and UMAP projections to assess heterogeneity within the clusters.

### Gene-set enrichment analysis:

*single-cell RNA sequencing.* Gene set enrichment analysis (GSEA) was performed to identify functional differences between cell types and experimental conditions using the *clusterProfiler* package.^[148]^ Ranked gene lists were generated using the *scoreMarkers* function from the *scran* package. The function evaluates marker genes based on two metrics: the area under the curve (AUC), a non-parametric measure quantifying a gene’s ability to distinguish cells in a cluster, and the median log-fold change, which robustly measures the effect size of differential expression. Genes were ranked by their AUC scores and sorted by median log-fold change to prioritize robust markers.

The gene lists were processed for enrichment analysis by converting Ensembl IDs to gene symbols using a curated annotation table to ensure consistency. GSEA was conducted using the GSEA function in *clusterProfiler* with Hallmark gene sets from the Molecular Signatures Database (*MSigDB*)^[149]^ as reference pathways, provided through the TERM2GENE argument. The analysis employed the following parameters: a minimum gene set size of 10, 10,000 permutations (nPermSimple) for statistical robustness, and an FDR-adjusted p-value cutoff of 0.05 to control for multiple testing. Rankings were based on the fold-change values derived from the *scoreMarkers* output. The pairings parameter in *scoreMarkers* was set to evaluate two key types of comparisons: (1) all co- or tri-component spheroids or tumoroids versus mono spheroids (2) OVCAR3 co- or tri-component tumoroids and cluster 11 versus OVCAR3 mono spheroids.

To complement *GSEA*, the *gseGO* function was applied to explore pathway enrichments based on Gene Ontology (GO) terms, with an ontology type set to “ALL” (Biological Process, Cellular Component, and Molecular Function). Parameters included a minimum gene set size of 10, a p-value cutoff of 0.05, and an FDR adjustment for multiple testing. Both analyses (Hallmark and GO) were repeated for all experimental conditions, with seed values fixed (1011011) for reproducibility.

Visualization of the results was performed using *ggplot2*,^[150]^ with NES plotted along the x-axis and pathways on the y-axis. Enrichment results were exported in tabular format, while pathway visualizations were saved as PDF files for reproducibility.

### Ligand-Receptor Communication Analysis:

#### single-cell RNA sequencing.

To investigate intercellular communication, we employed *CellCall*^[151]^ to calculate ligand-receptor (L-R) scores and *CellChat*^[71]^ to generate sender-receiver interaction networks.

*CellCall* was used to compute ligand-receptor scores across experimental conditions. Log-normalized gene expression data for each condition were input into the *CreateNichConObject* function with the following parameters: a minimum of three expressed features per cell, a scale factor of 1, and Homo sapiens as the reference organism. Ligand-receptor communication profiles were calculated using the *TransCommuProfile* function, which employs a Spearman correlation test to identify highly correlated ligand-receptor pairs. The statistical parameters included: 1) Correlation p-value threshold: 0.05, 2) Minimum correlation: 0.1, 3) FDR correction: p.adjust ≤ 0.05, 4) Top correlated targets: 1, and 5) Weighted median aggregation: threshold set at the 90th percentile (probs = 0.9).

The resulting L-R communication scores (expr_l_r_log2_scale slot) were extracted, combined across conditions, and visualized as a heatmap using *ggplot2*. The heatmap was faceted by sender-receiver combinations (e.g., OVCAR3 → MSC, MSC → OVCAR3, MSC → M2), highlighting communication scores across mono- and co- and tri-component tumoroids. Custom color gradients were applied to emphasize varying communication strengths, and the final visualization was saved as LR_score_comparison.pdf.

Complementary to *CellCall*, *CellChat* was used to visualize sender-receiver communication networks. The *CellChatDB.human* database was employed to identify overexpressed ligand-receptor pairs. Statistical significance for overexpressed interactions was determined using a truncated mean approach with a trimming threshold of 0.1. Communication probabilities were calculated using: 1) Interaction length: 200–300, and 2) Scaling factor: 0.01.

Aggregated communication networks were generated using the *aggregateNet* function. Sender-receiver communication networks were visualized as circle plots to depict the number of interactions and their relative strengths. These visualizations highlighted the signaling dynamics within and between cell populations under mono-spheroids and co- or tri-component tumoroids.

#### Statistical Analysis.

Experiments were carried out using three to five biological replicates per condition. Statistical analysis was performed using GraphPad Prism 10. When appropriate, one-way ANOVA and two-way ANOVA were used to test significant differences and post-hoc analyses were conducted thereafter. Significant results are indicated with symbols and a significance level in all data.

## Data Availability

The data reported in this article are available upon reasonable requests made to the corresponding authors.

## References

[1] Cortez A. J., Tudrej P., Kujawa K. A., Lisowska K. M., Cancer Chemother Pharmacol 2018, 81, 17. 29249039 10.1007/s00280-017-3501-8 PMC5754410

[2] Menon U., Gentry-Maharaj A., Burnell M., Singh N., Ryan A., Karpinskyj C., Carlino G., Taylor J., Massingham S. K., Raikou M., Kalsi J. K., Woolas R., Manchanda R., Arora R., Casey L., Dawnay A., Dobbs S., Leeson S., Mould T., Seif M. W., Sharma A., Williamson K., Liu Y., Fallowfield L., McGuire A. J., Campbell S., Skates S. J., Jacobs I. J., Parmar M., Lancet 2021, 397, 2182. 33991479 10.1016/S0140-6736(21)00731-5 PMC8192829

[3] Cascio S., Chandler C., Zhang L., Sinno S., Gao B., Onkar S., Bruno T. C., Vignali D. A. A., Mahdi H., Osmanbeyoglu H. U., Vlad A. M., Coffman L. G., Buckanovich R. J., Sci Adv 2021, 7, eabi5790. 34767446 10.1126/sciadv.abi5790 PMC8589308

[4] Coffman L. G., Choi Y. J., McLean K., Allen B. L., di Magliano M. P., Buckanovich R. J., Oncotarget 2016, 7, 6916. 26755648 10.18632/oncotarget.6870 PMC4872758

[5] Izar B., Tirosh I., Stover E. H., Wakiro I., Cuoco M. S., Alter I., Rodman C., Leeson R., Su M. J., Shah P., Iwanicki M., Walker S. R., Kanodia A., Melms J. C., Mei S., Lin J. R., Porter C. B. M., Slyper M., Waldman J., Jerby-Arnon L., Ashenberg O., Brinker T. J., Mills C., Rogava M., Vigneau S., Sorger P. K., Garraway L. A., Konstantinopoulos P. A., Liu J. F., Matulonis U., Johnson B. E., Rozenblatt-Rosen O., Rotem A., Regev A., Nat Med 2020, 26, 1271. 32572264 10.1038/s41591-020-0926-0 PMC7723336

[6] Raghavan S., Mehta P., Xie Y., Lei Y. L., Mehta G., J Immunother Cancer 2019, 7, 190. 31324218 10.1186/s40425-019-0666-1 PMC6642605

[7] Raghavan S., Snyder C. S., Wang A., McLean K., Zamarin D., Buckanovich R. J., Mehta G., Cancers (Basel) 2020, 12, DOI 10.3390/cancers120820631. PMC7464970 32726910

[8] Tothill R. W., V Tinker A., George J., Brown R., Fox S. B., Lade S., Johnson D. S., Trivett M. K., Etemadmoghadam D., Locandro B., Traficante N., Fereday S., Hung J. A., Chiew Y.-E., Haviv I., G. Australian Ovarian Cancer Study, Gertig D., deFazio A., Bowtell D. D. L., Clinical Cancer Research 2008, 14, 5198. 18698038 10.1158/1078-0432.CCR-08-0196

[9] Bell D., Berchuck A., Birrer M., Chien J., Cramer D. W., Dao F., Dhir R., DiSaia P., Gabra H., Glenn P., Godwin A. K., Gross J., Hartmann L., Huang M., Huntsman D. G., Iacocca M., Imielinski M., Kalloger S., Karlan B. Y., Levine D. A., Mills G. B., Morrison C., Mutch D., Olvera N., Orsulic S., Park K., Petrelli N., Rabeno B., Rader J. S., Sikic B. I., Smith-McCune K., Sood A. K., Bowtell D., Penny R., Testa J. R., Chang K., Creighton C. J., Dinh H. H., Drummond J. A., Fowler G., Gunaratne P., Hawes A. C., Kovar C. L., Lewis L. R., Morgan M. B., Newsham I. F., Santibanez J., Reid J. G., Trevino L. R., Wu Y. Q., Wang M., Muzny D. M., Wheeler D. A., Gibbs R. A., Getz G., Lawrence M. S., Cibulskis K., Sivachenko A. Y., Sougnez C., Voet D., Wilkinson J., Bloom T., Ardlie K., Fennell T., Baldwin J., Nichol R., Fisher S., Gabriel S., Lander E. S., Ding L., Fulton R. S., Koboldt D. C., McLellan M. D., Wylie T., Walker J., O’Laughlin M., Dooling D. J., Fulton L., Abbott R., Dees N. D., Zhang Q., Kandoth C., Wendl M., Schierding W., Shen D., Harris C. C., Schmidt H., Kalicki J., Delehaunty K. D., Fronick C. C., Demeter R., Cook L., Wallis J. W., Lin L., Magrini V. J., Hodges J. S., Eldred J. M., Smith S. M., Pohl C. S., Vandin F., Upfal E., Raphael B. J., Weinstock G. M., Mardis E. R., Wilson R. K., Meyerson M., Winckler W., Getz G., Verhaak R. G. W., Carter S. L., Mermel C. H., Saksena G., Nguyen H., Onofrio R. C., Lawrence M. S., Hubbard D., Gupta S., Crenshaw A., Ramos A. H., Ardlie K., Chin L., Protopopov A., Zhang J., Kim T. M., Perna I., Xiao Y., Zhang H., Ren G., Sathiamoorthy N., Park R. W., Lee E., Park P. J., Kucherlapati R., Absher D. M., Waite L., Sherlock G., Brooks J. D., Li J. Z., Xu J., Myers R. M., Laird P. W., Cope L., Herman J. G., Shen H., Weisenberger D. J., Noushmehr H., Pan F., Triche T. Jr, Berman B. P., Van Den Berg D. J., Buckley J., Baylin S. B., Spellman P. T., Purdom E., Neuvial P., Bengtsson H., Jakkula L. R., Durinck S., Han J., Dorton S., Marr H., Choi Y. G., Wang V., Wang N. J., Ngai J., Conboy J. G., Parvin B., Feiler H. S., Speed T. P., Gray J. W., Levine D. A., Socci N. D., Liang Y., Taylor B. S., Schultz N., Borsu L., Lash A. E., Brennan C., Viale A., Sander C., Ladanyi M., Hoadley K. A., Meng S., Du Y., Shi Y., Li L., Turman Y. J., Zang D., Helms E. B., Balu S., Zhou X., Wu J., Topal M. D., Hayes D. N., Perou C. M., Getz G., Voet D., Saksena G., Zhang J., Zhang H., Wu C. J., Shukla S., Cibulskis K., Lawrence M. S., Sivachenko A., Jing R., Park R. W., Liu Y., Park P. J., Noble M., Chin L., Carter H., Kim D., Samayoa J., Karchin R., Spellman P. T., Purdom E., Neuvial P., Bengtsson H., Durinck S., Han J., Korkola J. E., Heiser L. M., Cho R. J., Hu Z., Parvin B., Speed T. P., Gray J. W., Schultz N., Cerami E., Taylor B. S., Olshen A., Reva B., Antipin Y., Shen R., Mankoo P., Sheridan R., Ciriello G., Chang W. K., Bernanke J. A., Borsu L., Levine D. A., Ladanyi M., Sander C., Haussler D., Benz C. C., Stuart J. M., Benz S. C., Sanborn J. Z., Vaske C. J., Zhu J., Szeto C., Scott G. K., Yau C., Hoadley K. A., Du Y., Balu S., Hayes D. N., Perou C. M., Wilkerson M. D., Zhang N., Akbani R., Baggerly K. A., Yung W. K., Mills G. B., Weinstein J. N., Penny R., Shelton T., Grimm D., Hatfield M., Morris S., Yena P., Rhodes P., Sherman M., Paulauskis J., Millis S., Kahn A., Greene J. M., The N. Cancer Genome Atlas Research, group Disease working, sites tissue source, M. Genome sequencing centres: Baylor College of, I. Broad, L. Washington University in St, I. Cancer genome characterization centres: Broad Institute/Dana-Farber Cancer, S. Harvard Medical, U. HudsonAlpha Institute/Stanford, U. University of Southern California/Johns Hopkins, L. Lawrence Berkeley National, C. Memorial Sloan-Kettering Cancer, H. University of North Carolina at Chapel, I. Genome data analysis centres: Broad, U. Johns Hopkins, I. University of California Santa Cruz/Buck, M. D. A. C. C. The University of Texas, resource Biospecimen core, centre Data coordination, Nature 2011, 474, 609.21720365

[10] Konecny G. E., Wang C., Hamidi H., Winterhoff B., Kalli K. R., Dering J., Ginther C., Chen H. W., Dowdy S., Cliby W., Gostout B., Podratz K. C., Keeney G., Wang H. J., Hartmann L. C., Slamon D. J., Goode E. L., J Natl Cancer Inst 2014, 106, DOI 10.1093/jnci/dju249. PMC4271115 25269487

[11] Yang J. Y., Yoshihara K., Tanaka K., Hatae M., Masuzaki H., Itamochi H., Takano M., Ushijima K., Tanyi J. L., Coukos G., Lu Y., Mills G. B., Verhaak R. G., J Clin Invest 2013, 123, 3740. 23945238 10.1172/JCI68509 PMC3754259

[12] Verhaak R. G., Tamayo P., Yang J. Y., Hubbard D., Zhang H., Creighton C. J., Fereday S., Lawrence M., Carter S. L., Mermel C. H., Kostic A. D., Etemadmoghadam D., Saksena G., Cibulskis K., Duraisamy S., Levanon K., Sougnez C., Tsherniak A., Gomez S., Onofrio R., Gabriel S., Chin L., Zhang N., Spellman P. T., Zhang Y., Akbani R., Hoadley K. A., Kahn A., Köbel M., Huntsman D., Soslow R. A., Defazio A., Birrer M. J., Gray J. W., Weinstein J. N., Bowtell D. D., Drapkin R., Mesirov J. P., Getz G., Levine D. A., Meyerson M., J Clin Invest 2013, 123, 517. 23257362 10.1172/JCI65833 PMC3533304

[13] Doherty J. A., Peres L. C., Wang C., Way G. P., Greene C. S., Schildkraut J. M., Curr Epidemiol Rep 2017, 4, 211. 29226065 10.1007/s40471-017-0115-y PMC5718213

[14] Mei S., Chelmow D., Gecsi K., Barkley J., Barrows E., Brooks R., Huber-Keener K., Jeudy M., O’Hara J. S., Burke W., Obstet Gynecol 2023, 142, 196. 37348095 10.1097/AOG.0000000000005210 PMC10278570

[15] Davidson N. R., Barnard M. E., Hippen A. A., Campbell A., Johnson C. E., Way G. P., Dalley B. K., Berchuck A., Salas L. A., Peres L. C., Marks J. R., Schildkraut J. M., Greene C. S., Doherty J. A., Cancer Epidemiol Biomarkers Prev 2024, 33, 1114. 38780898 10.1158/1055-9965.EPI-24-0113 PMC11294000

[16] Talhouk A., George J., Wang C., Budden T., Tan T. Z., Chiu D. S., Kommoss S., Leong H. S., Chen S., Intermaggio M. P., Gilks B., Nazeran T. M., Volchek M., Elatre W., Bentley R. C., Senz J., Lum A., Chow V., Sudderuddin H., Mackenzie R., Leong S. C. Y., Liu G., Johnson D., Chen B., Group A., Alsop J., Banerjee S. N., Behrens S., Bodelon C., Brand A. H., Brinton L., Carney M. E., Chiew Y. E., Cushing-Haugen K. L., Cybulski C., Ennis D., Fereday S., Fortner R. T., Garcia-Donas J., Gentry-Maharaj A., Glasspool R., Goranova T., Greene C. S., Haluska P., Harris H. R., Hendley J., Hernandez B. Y., Herpel E., Jimenez-Linan M., Karpinskyj C., Kaufmann S. H., Keeney G. L., Kennedy C. J., Kobel M., Koziak J. M., Larson M. C., Lester J., Lewsley L. A., Lissowska J., Lubinski J., Luk H., Macintyre G., Mahner S., McNeish I. A., Menkiszak J., Nevins N., Osorio A., Oszurek O., Palacios J., Hinsley S., Pearce C. L., Pike M. C., Piskorz A. M., Ray-Coquard I., Rhenius V., Rodriguez-Antona C., Sharma R., Sherman M. E., De Silva D., Singh N., Sinn P., Slamon D., Song H., Steed H., Stronach E. A., Thompson P. J., Toloczko A., Trabert B., Traficante N., Tseng C. C., Widschwendter M., Wilkens L. R., Winham S. J., Winterhoff B., Beeghly-Fadiel A., Benitez J., Berchuck A., Brenton J. D., Brown R., Chang-Claude J., Chenevix-Trench G., deFazio A., Fasching P. A., Garcia M. J., Gayther S. A., Goodman M. T., Gronwald J., Henderson M. J., Karlan B. Y., Kelemen L. E., Menon U., Orsulic S., Pharoah P. D. P., Wentzensen N., Wu A. H., Schildkraut J. M., Rossing M. A., Konecny G. E., Huntsman D. G., Huang R. Y., Goode E. L., Ramus S. J., Doherty J. A., Bowtell D. D., Anglesio M. S., Clin Cancer Res 2020, 26, 5411. 32554541 10.1158/1078-0432.CCR-20-0103 PMC7572656

[17] Tan T. Z., Miow Q. H., Huang R. Y., Wong M. K., Ye J., Lau J. A., Wu M. C., Bin Abdul Hadi L. H., Soong R., Choolani M., Davidson B., Nesland J. M., Wang L. Z., Matsumura N., Mandai M., Konishi I., Goh B. C., Chang J. T., Thiery J. P., Mori S., EMBO Mol Med 2013, 5, 1051. 23666744 10.1002/emmm.201201823 PMC3721473

[18] Way G. P., Rudd J., Wang C., Hamidi H., Fridley B. L., Konecny G. E., Goode E. L., Greene C. S., Doherty J. A., G3 (Bethesda) 2016, 6, 4097. 27729437 10.1534/g3.116.033514 PMC5144978

[19] Hornburg M., Desbois M., Lu S., Guan Y., Lo A. A., Kaufman S., Elrod A., Lotstein A., DesRochers T. M., Munoz-Rodriguez J. L., Wang X., Giltnane J., Mayba O., Turley S. J., Bourgon R., Daemen A., Wang Y., Cancer Cell 2021, 39, 928. 33961783 10.1016/j.ccell.2021.04.004

[20] Olalekan S., Xie B., Back R., Eckart H., Basu A., Cell Rep 2021, 35, 109165. 34038734 10.1016/j.celrep.2021.109165

[21] Wang Y., Xie H., Chang X., Hu W., Li M., Li Y., Liu H., Cheng H., Wang S., Zhou L., Shen D., Dou S., Ma R., Mao Y., Zhu H., Zhang X., Zheng Y., Ye X., Wen L., Kee K., Cui H., Tang F., Cancer Res 2022, 82, 3903. 35969151 10.1158/0008-5472.CAN-21-3819 PMC9627134

[22] Cheng H., Wang Z., Fu L., Xu T., Front Oncol 2019, 9, 421. 31192126 10.3389/fonc.2019.00421 PMC6540821

[23] Duluc D., Delneste Y., Tan F., Moles M. P., Grimaud L., Lenoir J., Preisser L., Anegon I., Catala L., Ifrah N., Descamps P., Gamelin E., Gascan H., Hebbar M., Jeannin P., Blood 2007, 110, 4319. 17848619 10.1182/blood-2007-02-072587

[24] Hagemann T., Wilson J., Burke F., Kulbe H., Li N. F., Pluddemann A., Charles K., Gordon S., Balkwill F. R., J Immunol 2006, 176, 5023. 16585599 10.4049/jimmunol.176.8.5023

[25] Liu L., Wang X., Li X., Wu X., Tang M., Wang X., Oncol Rep 2018, 39, 818. 29251331 10.3892/or.2017.6148

[26] Ning Y., Cui Y., Li X., Cao X., Chen A., Xu C., Cao J., Luo X., Biomed Pharmacother 2018, 103, 262. 29656182 10.1016/j.biopha.2018.04.022

[27] Steitz A. M., Steffes A., Finkernagel F., Unger A., Sommerfeld L., Jansen J. M., Wagner U., Graumann J., Müller R., Reinartz S., Cell Death Dis 2020, 11, 249. 32312959 10.1038/s41419-020-2438-8 PMC7171168

[28] Zhu X., Shen H., Yin X., Yang M., Wei H., Chen Q., Feng F., Liu Y., Xu W., Li Y., J Exp Clin Cancer Res 2019, 38, 81. 30770776 10.1186/s13046-019-1095-1 PMC6377760

[29] Horst E. N., Bregenzer M. E., Mehta P., Snyder C. S., Repetto T., Yang-Hartwich Y., Mehta G., Acta Biomater 2021, 132, 401. 33940195 10.1016/j.actbio.2021.04.041 PMC8969826

[30] Kopper O., de Witte C. J., Lõhmussaar K., Valle-Inclan J. E., Hami N., Kester L., V Balgobind A., Korving J., Proost N., Begthel H., van Wijk L. M., Revilla S. A., Theeuwsen R., van de Ven M., van Roosmalen M. J., Ponsioen B., Ho V. W. H., Neel B. G., Bosse T., Gaarenstroom K. N., Vrieling H., Vreeswijk M. P. G., van Diest P. J., Witteveen P. O., Jonges T., Bos J. L., van Oudenaarden A., Zweemer R. P., Snippert H. J. G., Kloosterman W. P., Clevers H., Nat Med 2019, 25, 838. 31011202 10.1038/s41591-019-0422-6

[31] Chan W. S., Mo X., Ip P. P. C., Tse K. Y., Cancer Med 2023, 12, 19714. 37776168 10.1002/cam4.6521 PMC10587945

[32] de Witte C. J., Espejo Valle-Inclan J., Hami N., Lõhmussaar K., Kopper O., Vreuls C. P. H., Jonges G. N., van Diest P., Nguyen L., Clevers H., Kloosterman W. P., Cuppen E., Snippert H. J. G., Zweemer R. P., Witteveen P. O., Stelloo E., Cell Rep 2020, 31, 107762. 32553164 10.1016/j.celrep.2020.107762

[33] Nanki Y., Chiyoda T., Hirasawa A., Ookubo A., Itoh M., Ueno M., Akahane T., Kameyama K., Yamagami W., Kataoka F., Aoki D., Sci Rep 2020, 10, 12581. 32724113 10.1038/s41598-020-69488-9 PMC7387538

[34] Senkowski W., Gall-Mas L., Falco M. M., Li Y., Lavikka K., Kriegbaum M. C., Oikkonen J., Bulanova D., Pietras E. J., Voßgröne K., Chen Y.-J., Erkan E. P., Dai J., Lundgren A., Grønning Høg M. K., Larsen I. M., Lamminen T., Kaipio K., Huvila J., Virtanen A., Engelholm L., Christiansen P., Santoni-Rugiu E., Huhtinen K., Carpén O., Hynninen J., Hautaniemi S., Vähärautio A., Wennerberg K., Dev Cell 2023, 58, 1106. 37148882 10.1016/j.devcel.2023.04.012 PMC10281085

[35] Domcke S., Sinha R., Levine D. A., Sander C., Schultz N., Nat Commun 2013, 4, 2126. 23839242 10.1038/ncomms3126 PMC3715866

[36] Kurnit K. C., Fleming G. F., Lengyel E., Obstet Gynecol 2021, 137, 108. 33278287 10.1097/AOG.0000000000004173 PMC7737875

[37] Kuroki L., Guntupalli S. R., Bmj 2020, 371, m3773. 33168565 10.1136/bmj.m3773

[38] Richardson D. L., Eskander R. N., O’Malley D. M., JAMA Oncol 2023, 9, 851. 37079311 10.1001/jamaoncol.2023.0197

[39] Bicaku E., Xiong Y., Marchion D. C., Chon H. S., Stickles X. B., Chen N., Judson P. L., Hakam A., Gonzalez-Bosquet J., Wenham R. M., Apte S. M., Fulp W., Cubitt C. L., Chen D. T., Lancaster J. M., Br J Cancer 2012, 106, 1967. 22596241 10.1038/bjc.2012.207 PMC3388569

[40] Bregenzer M., Horst E., Mehta P., Snyder C., Repetto T., Mehta G., Methods Mol Biol 2022, 2424, 217. 34918298 10.1007/978-1-0716-1956-8_15 PMC10602930

[41] Bregenzer M. E., Horst E. N., Mehta P., Novak C. M., Repetto T., Mehta G., Cancers (Basel) 2019, 11, DOI 10.3390/cancers11071008. 31323899 PMC6679114

[42] Mehta P., Novak C., Raghavan S., Ward M., Mehta G., Methods Mol Biol 2018, 1692, 61. 28986887 10.1007/978-1-4939-7401-6_6 PMC5925737

[43] Iżycka N., Zaborowski M. P., Ciecierski Ł., Jaz K., Szubert S., Miedziarek C., Rezler M., Piątek-Bajan K., Synakiewicz A., Jankowska A., Figlerowicz M., Sterzyńska K., Nowak-Markwitz E., Int J Mol Sci 2023, 24, DOI 10.3390/ijms241612746. 37628927 PMC10454196

[44] Zhou J., Du Y., Lu Y., Luan B., Xu C., Yu Y., Zhao H., Front Oncol 2019, 9, 802. 31497537 10.3389/fonc.2019.00802 PMC6712994

[45] Zhang J., Yuan B., Zhang H., Li H., Oncol Lett 2019, 17, 5351. 31186752 10.3892/ol.2019.10221 PMC6507388

[46] Motohara T., Fujimoto K., Tayama S., Narantuya D., Sakaguchi I., Tashiro H., Katabuchi H., Obstet Gynecol 2016, 127, 1003. 27159753 10.1097/AOG.0000000000001420

[47] Gao M. Q., Choi Y. P., Kang S., Youn J. H., Cho N. H., Oncogene 2010, 29, 2672. 20190812 10.1038/onc.2010.35

[48] Burgos-Ojeda D., Wu R., McLean K., Chen Y. C., Talpaz M., Yoon E., Cho K. R., Buckanovich R. J., Mol Cancer Ther 2015, 14, 1717. 25969154 10.1158/1535-7163.MCT-14-0607 PMC4496272

[49] Nakamura K., Terai Y., Tanabe A., Ono Y. J., Hayashi M., Maeda K., Fujiwara S., Ashihara K., Nakamura M., Tanaka Y., Tanaka T., Tsunetoh S., Sasaki H., Ohmichi M., Oncol Rep 2017, 37, 3189. 28440503 10.3892/or.2017.5583 PMC5442399

[50] V Connor E., Saygin C., Braley C., Wiechert A. C., Karunanithi S., Crean-Tate K., Abdul-Karim F. W., Michener C. M., Rose P. G., Lathia J. D., Reizes O., J Ovarian Res 2019, 12, 112. 31735168 10.1186/s13048-019-0590-5 PMC6858973

[51] Lu J. W., Chang J. G., Yeh K. T., Chen R. M., Tsai J. J., Hu R. M., Acta Histochem 2011, 113, 833. 21272924 10.1016/j.acthis.2011.01.001

[52] Yang Y., Otte A., Hass R., Stem Cells Dev 2015, 24, 1205. 25525832 10.1089/scd.2014.0413 PMC4425222

[53] Curley M. D., Therrien V. A., Cummings C. L., Sergent P. A., Koulouris C. R., Friel A. M., Roberts D. J., V Seiden M., Scadden D. T., Rueda B. R., Foster R., Stem Cells 2009, 27, 2875. 19816957 10.1002/stem.236

[54] Silva I. A., Bai S., McLean K., Yang K., Griffith K., Thomas D., Ginestier C., Johnston C., Kueck A., Reynolds R. K., Wicha M. S., Buckanovich R. J., Cancer Res 2011, 71, 3991. 21498635 10.1158/0008-5472.CAN-10-3175 PMC3107359

[55] Zhou Q., Chen A., Song H., Tao J., Yang H., Zuo M., Int J Clin Exp Med 2015, 8, 3080. 26064196 PMC4443030

[56] Raghavan S., Mehta P., Ward M. R., Bregenzer M. E., Fleck E. M. A., Tan L., McLean K., Buckanovich R. J., Mehta G., Clin Cancer Res 2017, 23, 6934. 28814433 10.1158/1078-0432.CCR-17-0133 PMC6330017

[57] Ward Rashidi M. R., Mehta P., Bregenzer M., Raghavan S., Fleck E. M., Horst E. N., Harissa Z., Ravikumar V., Brady S., Bild A., Rao A., Buckanovich R. J., Mehta G., Neoplasia 2019, 21, 822. 31299607 10.1016/j.neo.2019.06.005 PMC6624324

[58] Meng E., Mitra A., Tripathi K., Finan M. A., Scalici J., McClellan S., Madeira da Silva L., Reed E., Shevde L. A., Palle K., Rocconi R. P., PLoS One 2014, 9, e107142. 25216266 10.1371/journal.pone.0107142 PMC4162571

[59] Choi Y. J., Ingram P. N., Yang K., Coffman L., Iyengar M., Bai S., Thomas D. G., Yoon E., Buckanovich R. J., Proc Natl Acad Sci U S A 2015, 112, E6882. 26621735 10.1073/pnas.1507899112 PMC4687560

[60] Richardson D. L., Moore K. N., Vergote I., Gilbert L., Martin L. P., Mantia-Smaldone G. M., Castro C. M., Provencher D., Matulonis U. A., Stec J., Wang Y., Method M., O’Malley D. M., Gynecol Oncol 2024, 185, 186. 38447347 10.1016/j.ygyno.2024.01.045

[61] Yousefi M., Dehghani S., Nosrati R., Ghanei M., Salmaninejad A., Rajaie S., Hasanzadeh M., Pasdar A., Cell Oncol (Dordr) 2020, 43, 515. 32418122 10.1007/s13402-020-00513-9 PMC12990730

[62] Tan D. S. P., Agarwal R., Kaye S. B., Lancet Oncol 2006, 7, 925. 17081918 10.1016/S1470-2045(06)70939-1

[63] Ritch S. J., Telleria C. M., Front Endocrinol (Lausanne) 2022, 13, 886533. 35574025 10.3389/fendo.2022.886533 PMC9096207

[64] Davidowitz R. A., Selfors L. M., Iwanicki M. P., Elias K. M., Karst A., Piao H., Ince T. A., Drage M. G., Dering J., Konecny G. E., Matulonis U., Mills G. B., Slamon D. J., Drapkin R., Brugge J. S., J Clin Invest 2014, 124, 2611. 24762435 10.1172/JCI69815 PMC4038562

[65] Augimeri G., Gonzalez M. E., Paolì A., Eido A., Choi Y., Burman B., Djomehri S., Karthikeyan S. K., Varambally S., Buschhaus J. M., Chen Y. C., Mauro L., Bonofiglio D., Nesvizhskii A. I., Luker G. D., Andò S., Yoon E., Kleer C. G., JCI Insight 2023, 8, DOI 10.1172/jci.insight.164216. PMC10561721 37607007

[66] Chen Y. C., Gonzalez M. E., Burman B., Zhao X., Anwar T., Tran M., Medhora N., Hiziroglu A. B., Lee W., Cheng Y. H., Choi Y., Yoon E., Kleer C. G., Cell Rep 2019, 27, 3916. 31242423 10.1016/j.celrep.2019.05.084 PMC6657699

[67] Gast C. E., Silk A. D., Zarour L., Riegler L., Burkhart J. G., Gustafson K. T., Parappilly M. S., Roh-Johnson M., Goodman J. R., Olson B., Schmidt M., Swain J. R., Davies P. S., Shasthri V., Iizuka S., Flynn P., Watson S., Korkola J., Courtneidge S. A., Fischer J. M., Jaboin J., Billingsley K. G., Lopez C. D., Burchard J., Gray J., Coussens L. M., Sheppard B. C., Wong M. H., Sci Adv 2018, 4, eaat7828. 30214939 10.1126/sciadv.aat7828 PMC6135550

[68] Gironda D. J., Bergan R. C., Alpaugh R. K., Danila D. C., Chuang T. L., Hurtado B. Y., Ho T., Adams D. L., Cancers (Basel) 2023, 15, DOI 10.3390/cancers15143725. PMC10378487 37509385

[69] Manjunath Y., Mitchem J. B., Suvilesh K. N., Avella D. M., Kimchi E. T., Staveley-O’Carroll K. F., Deroche C. B., Pantel K., Li G., Kaifi J. T., J Thorac Oncol 2020, 15, 1460. 32416323 10.1016/j.jtho.2020.04.034

[70] A. P. C. Ruano, A. P. Gadelha Guimarães, A. C. Braun, B. Flores, M. S. Tariki, E. A. Abdallah, J. A. Torres, D. N. Nunes, B. Tirapelli, V. C. C. de Lima, M. F. Fanelli, P. E. Colombo, A. da Costa, C. Alix-Panabières, L. T. D. Chinen, Int J Mol Sci 2022, 23, DOI 10.3390/ijms232314687. PMC9740150 36499015

[71] Jin S., Guerrero-Juarez C. F., Zhang L., Chang I., Ramos R., Kuan C.-H., Myung P., V Plikus M., Nie Q., Nat Commun 2021, 12, 1088. 33597522 10.1038/s41467-021-21246-9 PMC7889871

[72] Zhang Y., Liu T., Hu X., Wang M., Wang J., Zou B., Tan P., Cui T., Dou Y., Ning L., Huang Y., Rao S., Wang D., Zhao X., Nucleic Acids Res 2021, 49, 8520. 34331449 10.1093/nar/gkab638 PMC8421219

[73] Pearce O. M. T., Delaine-Smith R. M., Maniati E., Nichols S., Wang J., Böhm S., Rajeeve V., Ullah D., Chakravarty P., Jones R. R., Montfort A., Dowe T., Gribben J., Jones J. L., Kocher H. M., Serody J. S., Vincent B. G., Connelly J., Brenton J. D., Chelala C., Cutillas P. R., Lockley M., Bessant C., Knight M. M., Balkwill F. R., Cancer Discov 2018, 8, 304. 29196464 10.1158/2159-8290.CD-17-0284 PMC5837004

[74] Rockey D. C., Weymouth N., Shi Z., PLoS One 2013, 8, DOI 10.1371/journal.pone.0077166. PMC3812165 24204762

[75] Link P. A., Choi K. M., Diaz Espinosa A. M., Jones D. L., Gao A. Y., Haak A. J., Tschumperlin D. J., Am J Physiol Lung Cell Mol Physiol 2022, 322, DOI 10.1152/AJPLUNG.00210.2021. PMC8721907 34755530

[76] Hara M., Yokota K., Saito T., Kobayakawa K., Kijima K., Yoshizaki S., Okazaki K., Yoshida S., Matsumoto Y., Harimaya K., Nakashima Y., Okada S., Journal of Bone and Joint Surgery 2018, 100, DOI 10.2106/JBJS.17.01230. 30106825

[77] Lin S. C., Liao Y. C., Chen P. M., Yang Y. Y., Wang Y. H., Tung S. L., Chuang C. M., Sung Y. W., Jang T. H., Chuang S. E., Wang L. H., J Biomed Sci 2022, 29, DOI 10.1186/s12929-022-00888-x. PMC9784270 36550569

[78] Puttock E. H., Tyler E. J., Manni M., Maniati E., Butterworth C., Burger Ramos M., Peerani E., Hirani P., Gauthier V., Liu Y., Maniscalco G., Rajeeve V., Cutillas P., Trevisan C., Pozzobon M., Lockley M., Rastrick J., Läubli H., White A., Pearce O. M. T., Nat Commun 2023, 14, 2514. 37188691 10.1038/s41467-023-38093-5 PMC10185550

[79] Zhang K., Erkan E. P., Jamalzadeh S., Dai J., Andersson N., Kaipio K., Lamminen T., Mansuri N., Huhtinen K., Carpén O., Hietanen S., Oikkonen J., Hynninen J., Virtanen A., Häkkinen A., Hautaniemi S., Vähärautio A., Sci Adv 2022, 8, eabm1831. 35196078 10.1126/sciadv.abm1831 PMC8865800

[80] Gentles A. J., Newman A. M., Liu C. L., V Bratman S., Feng W., Kim D., Nair V. S., Xu Y., Khuong A., Hoang C. D., Diehn M., West R. B., Plevritis S. K., Alizadeh A. A., Nat Med 2015, 21, 938. 26193342 10.1038/nm.3909 PMC4852857

[81] Győrffy B., Br J Pharmacol 2024, 181, 362. 37783508 10.1111/bph.16257

[82] Győrffy B., Geroscience 2023, 45, DOI 10.1007/s11357-023-00742-4. PMC10400493 36856946

[83] Hu Y., Taylor-Harding B., Raz Y., Haro M., Recouvreux M. S., Taylan E., Lester J., Millstein J., Walts A. E., Karlan B. Y., Orsulic S., Front Cell Dev Biol 2020, 8, DOI 10.3389/fcell.2020.00647. PMC7380132 32766252

[84] Samain R., Brunel A., Douché T., Fanjul M., Cassant-Sourdy S., Rochotte J., Cros J., Neuzillet C., Raffenne J., Duluc C., Perraud A., Nigri J., Gigoux V., Bieche I., Ponzo M., Carpentier G., Cascone I., Tomasini R., Schmid H. A., Mathonnet M., Nicolle R., Bousquet M. P., Martineau Y., Pyronnet S., Jean C., Bousquet C., Cell Mol Gastroenterol Hepatol 2021, 11, 1405. 33482394 10.1016/j.jcmgh.2021.01.008 PMC8024982

[85] Wolfe A. R., Trenton N. J., Debeb B. G., Larson R., Ruffell B., Chu K., Hittelman W., Diehl M., Reuben J. M., Ueno N. T., Woodward W. A., Oncotarget 2016, 7, 82482. 27756885 10.18632/oncotarget.12694 PMC5347707

[86] Li W., Zhang X., Wu F., Zhou Y., Bao Z., Li H., Zheng P., Zhao S., Cell Death Dis 2019, 10, 918. 31801938 10.1038/s41419-019-2131-y PMC6892854

[87] Babazadeh S., Nassiri S. M., Siavashi V., Sahlabadi M., Hajinasrollah M., Zamani-Ahmadmahmudi M., Cell Mol Biol Lett 2021, 26, 30. 34174813 10.1186/s11658-021-00273-w PMC8236206

[88] Biswas S., Mandal G., Roy Chowdhury S., Purohit S., Payne K. K., Anadon C., Gupta A., Swanson P., Yu X., Conejo-Garcia J. R., Bhattacharyya A., J Immunol 2019, 203, 3447. 31704881 10.4049/jimmunol.1900692 PMC6994919

[89] Bregenzer M. E., Davis C., Horst E. N., Mehta P., Novak C. M., Raghavan S., Snyder C. S., Mehta G., J Vis Exp 2019, DOI 10.3791/59696. PMC9894675 31329171

[90] Raghavan S., Mehta P., Horst E. N., Ward M. R., Rowley K. R., Mehta G., Oncotarget 2016, DOI 10.18632/oncotarget.7659. PMC4941362 26918944

[91] Raghavan S., Ward M. R., Rowley K. R., Wold R. M., Takayama S., Buckanovich R. J., Mehta G., Gynecol Oncol 2015, 138, 181. 25913133 10.1016/j.ygyno.2015.04.014 PMC4480341

[92] Świerczewska M., Sterzyńska K., Ruciński M., Andrzejewska M., Nowicki M., Januchowski R., Biomed Pharmacother 2023, 165, 115152. 37442067 10.1016/j.biopha.2023.115152

[93] McLean K., Tan L., Bolland D. E., Coffman L. G., Peterson L. F., Talpaz M., Neamati N., Buckanovich R. J., Oncogene 2019, 38, 1576. 30305729 10.1038/s41388-018-0523-6 PMC6374186

[94] Steg A. D., Bevis K. S., Katre A. A., Ziebarth A., Dobbin Z. C., Alvarez R. D., Zhang K., Conner M., Landen C. N., Clin Cancer Res 2012, 18, 869. 22142828 10.1158/1078-0432.CCR-11-2188 PMC3271164

[95] Landen C. N. Jr., Goodman B., Katre A. A., Steg A. D., Nick A. M., Stone R. L., Miller L. D., Mejia P. V, Jennings N. B., Gershenson D. M., Bast R. C. Jr., Coleman R. L., Lopez-Berestein G., Sood A. K., Mol Cancer Ther 2010, 9, 3186. 20889728 10.1158/1535-7163.MCT-10-0563 PMC3005138

[96] Alvero A. B., Chen R., Fu H. H., Montagna M., Schwartz P. E., Rutherford T., Silasi D. A., Steffensen K. D., Waldstrom M., Visintin I., Mor G., Cell Cycle 2009, 8, 158. 19158483 10.4161/cc.8.1.7533 PMC3041590

[97] Kurrey N. K., Jalgaonkar S. P., V Joglekar A., Ghanate A. D., Chaskar P. D., Doiphode R. Y., Bapat S. A., Stem Cells 2009, 27, 2059. 19544473 10.1002/stem.154

[98] Deng J., Wang L., Chen H., Hao J., Ni J., Chang L., Duan W., Graham P., Li Y., Oncotarget 2016, 7, 55771. 27304054 10.18632/oncotarget.9908 PMC5342453

[99] Nwani N. G., Condello S., Wang Y., Swetzig W. M., Barber E., Hurley T., Matei D., Cancers (Basel) 2019, 11, DOI 10.3390/cancers11040502. PMC6521036 30965686

[100] Caminear M. W., Harrington B. S., Kamdar R. D., Kruhlak M. J., Annunziata C. M., Front Oncol 2022, 12, 762820. 35372040 10.3389/fonc.2022.762820 PMC8967967

[101] Nagare R. P., Sneha S., Krishnapriya S., Ramachandran B., Murhekar K., Vasudevan S., Shabna A., Ganesan T. S., Exp Cell Res 2020, 392, 112009. 32305326 10.1016/j.yexcr.2020.112009

[102] Melzer C., von der Ohe J., Lehnert H., Ungefroren H., Hass R., Mol Cancer 2017, 16, 28. 28148265 10.1186/s12943-017-0595-x PMC5286787

[103] Sainz B. Jr., Carron E., Vallespinós M., Machado H. L., Mediators Inflamm 2016, 2016, 9012369. 26980947 10.1155/2016/9012369 PMC4769767

[104] Long L., Hu Y., Long T., Lu X., Tuo Y., Li Y., Ke Z., J Immunother Cancer 2021, 9, DOI 10.1136/jitc-2021-003973. PMC8718465 34969774

[105] So K. A., Min K. J., Hong J. H., Lee J. K., Int J Oncol 2015, 47, 1451. 26316317 10.3892/ijo.2015.3122

[106] Zeng X.-Y., Xie H., Yuan J., Jiang X.-Y., Yong J.-H., Zeng D., Dou Y.-Y., Xiao S.-S., Cancer Biol Ther 2019, 20, 956. 31062668 10.1080/15384047.2018.1564567 PMC6606001

[107] Tan T. Z., Miow Q. H., Miki Y., Noda T., Mori S., Huang R. Y., Thiery J. P., EMBO Mol Med 2014, 6, 1279. 25214461 10.15252/emmm.201404208 PMC4287932

[108] Arend R. C., Londono-Joshi A. I., Straughn J. M. Jr., D. J. Buchsbaum, Gynecol Oncol 2013, 131, 772. 24125749 10.1016/j.ygyno.2013.09.034

[109] Asem M., Young A. M., Oyama C., Claure A. De La Zerda, Y. Liu, J. Yang, T. S. Hilliard, J. Johnson, E. I. Harper, I. Guldner, S. Zhang, T. Page-Mayberry, W. J. Kaliney, M. S. Stack, Cancer Res 2020, 80, 1156. 31932454 10.1158/0008-5472.CAN-19-1601 PMC8245162

[110] Azimian-Zavareh V., Dehghani-Ghobadi Z., Ebrahimi M., Mirzazadeh K., Nazarenko I., Hossein G., Sci Rep 2021, 11, 5885. 33723319 10.1038/s41598-021-85356-6 PMC7970989

[111] Etzerodt A., Moulin M., Doktor T. K., Delfini M., Mossadegh-Keller N., Bajenoff M., Sieweke M. H., Moestrup S. K., Auphan-Anezin N., Lawrence T., J Exp Med 2020, 217, DOI 10.1084/jem.20191869. PMC7144521 31951251

[112] Fang Y., Xiao X., Wang J., Dasari S., Pepin D., Nephew K. P., Zamarin D., Mitra A. K., NPJ Precis Oncol 2024, 8, 7. 38191909 10.1038/s41698-023-00495-5 PMC10774407

[113] Ford C. E., Punnia-Moorthy G., Henry C. E., Llamosas E., Nixdorf S., Olivier J., Caduff R., Ward R. L., Heinzelmann-Schwarz V., Gynecol Oncol 2014, 134, 338. 24924122 10.1016/j.ygyno.2014.06.004

[114] Grither W. R., Baker B., Morikis V. A., Ilagan M. X. G., Fuh K. C., Longmore G. D., Mol Cancer Res 2024, 22, 495. 38334461 10.1158/1541-7786.MCR-23-0616 PMC11065611

[115] Peng C., Zhang X., Yu H., Wu D., Zheng J., Int J Gynecol Cancer 2011, 21, 280. 21270611 10.1097/IGC.0b013e31820aaadb

[116] Dausinas P., Pulakanti K., Rao S., Cole J. M., Dahl R., Cowden Dahl K. D., Gene 2020, 738, 144458. 32061921 10.1016/j.gene.2020.144458 PMC7384259

[117] Zong X., Wang W., Ozes A., Fang F., Sandusky G. E., Nephew K. P., Cancer Res 2020, 80, 4371. 32816909 10.1158/0008-5472.CAN-20-0458 PMC7572866

[118] Mishra P. J., Mishra P. J., Humeniuk R., Medina D. J., Alexe G., Mesirov J. P., Ganesan S., Glod J. W., Banerjee D., Cancer Res 2008, 68, 4331. 18519693 10.1158/0008-5472.CAN-08-0943 PMC2725025

[119] Spaeth E. L., Dembinski J. L., Sasser A. K., Watson K., Klopp A., Hall B., Andreeff M., Marini F., PLoS One 2009, 4, e4992. 19352430 10.1371/journal.pone.0004992 PMC2661372

[120] Calon A., Lonardo E., Berenguer-Llergo A., Espinet E., Hernando-Momblona X., Iglesias M., Sevillano M., Palomo-Ponce S., V Tauriello D., Byrom D., Cortina C., Morral C., Barceló C., Tosi S., Riera A., Attolini C. S., Rossell D., Sancho E., Batlle E., Nat Genet 2015, 47, 320. 25706628 10.1038/ng.3225

[121] Wessolly M., Mairinger E., Borchert S., Bankfalvi A., Mach P., Schmid K. W., Kimmig R., Buderath P., Mairinger F. D., Front Oncol 2022, 12, 798680. 35311102 10.3389/fonc.2022.798680 PMC8927667

[122] Turcotte M., Spring K., Pommey S., Chouinard G., Cousineau I., George J., Chen G. M., Gendoo D. M., Haibe-Kains B., Karn T., Rahimi K., Le Page C., Provencher D., Mes-Masson A. M., Stagg J., Cancer Res 2015, 75, 4494. 26363007 10.1158/0008-5472.CAN-14-3569

[123] Deng Y., Tan Y., Zhou D., Bai Y., Cao T., Zhong C., Huang W., Ou Y., Guo L., Liu Q., Yin D., Chen L., Luo X., Sun D., Sheng X., Front Immunol 2022, 13, 923194. 35935940 10.3389/fimmu.2022.923194 PMC9354882

[124] Arafat H., Lazar M., Salem K., Chipitsyna G., Gong Q., Pan T. C., Zhang R. Z., Yeo C. J., Chu M. L., Surgery 2011, 150, DOI 10.1016/j.surg.2011.05.011. PMC3163121 21719059

[125] Li X., Jin Y., Xue J., Int J Gen Med 2024, *Volume* 17, 1773. 38711825 10.2147/IJGM.S463649 PMC11073151

[126] Svoronos C., Tsoulfas G., Souvatzi M., Chatzitheoklitos E., Ann Hepatobiliary Pancreat Surg 2020, 24, DOI 10.14701/ahbps.2020.24.1.52. PMC7061042 32181429

[127] Xu H., Xue S., Sun Y., Ma J., Li S., Wang Y., Mao T., Ge W., Yue M., Shentu D., Lu W., Wang Y., Hu J., Cui J., Zhang X., Cai L., Wang Y., Wang L., J Immunother Cancer 2025, 13, e010029. 39762079 10.1136/jitc-2024-010029 PMC11749327

[128] Lei X., Chen G., Li J., Wen W., Gong J., Fu J., PeerJ 2021, 9, DOI 10.7717/peerj.12141. PMC8428264 34567847

[129] Ashok G., Miryala S. K., Saju M. T., Anbarasu A., Ramaiah S., Molecular Genetics and Genomics 2022, 297, DOI 10.1007/s00438-022-01943-w. 35982245

[130] Wu J., Liu J., Wei X. Q., Yu Q., Niu X. H., Tang S. H., Song L., J Enzyme Inhib Med Chem 2019, 34, DOI 10.1080/14756366.2018.1484734. PMC6327995 30734598

[131] Owusu-Ansah K. G., Song G., Chen R., Edoo M. I. A., Li J., Chen B., Wu J., Zhou L., Xie H., Jiang D., Zheng S., Int J Oncol 2019, 55, DOI 10.3892/ijo.2019.4825. PMC6615918 31268154

[132] Li X., Sun X., Kan C., Chen B., Qu N., Hou N., Liu Y., Han F., Pathol Res Pract 2022, 236, DOI 10.1016/j.prp.2022.154013. 35816922

[133] Chen C., Ye L., Yi J., Liu T., Li Z., Breast Cancer Res Treat 2023, 201, DOI 10.1007/s10549-023-07032-9. 37458908

[134] Zhang X. X., Luo J. H., Wu L. Q., Front Genet 2022, 13, DOI 10.3389/fgene.2022.913659.

[135] Assidicky R., Tokat U. M., Tarman I. O., Saatci O., Ersan P. G., Raza U., Ogul H., Riazalhosseini Y., Can T., Sahin O., Breast Cancer Res Treat 2022, 193, DOI 10.1007/s10549-022-06569-5. PMC9389626 35338412

[136] Wei L. Y., Zhang X. J., Wang L., Hu L. N., Zhang X. D., Li L., Gao J. N., Onco Targets Ther 2020, 13, DOI 10.2147/OTT.S256818. PMC7342558 32753890

[137] Bregenzer M., Horst E., Mehta P., Snyder C., Repetto T., Mehta G., in Methods in Molecular Biology, 2022.

[138] Burkhard K. M., Mehta G., in Methods in Molecular Biology, 2024.

[139] Melsted P., Booeshaghi A. S., Liu L., Gao F., Lu L., (Joseph) Min K. H., da Veiga Beltrame E., Hjörleifsson K. E., Gehring J., Pachter L., Nat Biotechnol 2021, 39, DOI 10.1038/s41587-021-00870-2. 33795888

[140] Lun A. T. L., Riesenfeld S., Andrews T., Dao T. P., Gomes T., Marioni J. C., Genome Biol 2019, 20, DOI 10.1186/s13059-019-1662-y. PMC6431044 30902100

[141] McCarthy D. J., Campbell K. R., Lun A. T. L., Wills Q. F., Bioinformatics 2017, 33, DOI 10.1093/bioinformatics/btw777. PMC5408845 28088763

[142] Lun A. T. L., McCarthy D. J., Marioni J. C., F1000Res 2016, 5, DOI 10.12688/f1000research.9501.2. PMC5112579 27909575

[143] Germain P. L., Robinson M. D., Lun A., Garcia Meixide C., Macnair W., F1000Res 2022, 10, DOI 10.12688/f1000research.73600.2. PMC9204188 35814628

[144] Haghverdi L., Lun A. T. L., Morgan M. D., Marioni J. C., Nat Biotechnol 2018, 36, DOI 10.1038/nbt.4091. PMC6152897 29608177

[145] Lun A., Hicks S., Courbayre B., Borman T., Lahti L., 2024, DOI 10.18129/B9.bioc.bluster.

[146] Alquicira-Hernandez J., Powell J. E., Bioinformatics 2021, 37, DOI 10.1093/bioinformatics/btab003. 33459785

[147] Aran D., Looney A. P., Liu L., Wu E., Fong V., Hsu A., Chak S., Naikawadi R. P., Wolters P. J., Abate A. R., Butte A. J., Bhattacharya M., Nat Immunol 2019, 20, DOI 10.1038/s41590-018-0276-y. PMC6340744 30643263

[148] Yu G., Wang L. G., Han Y., He Q. Y., OMICS 2012, 16, DOI 10.1089/omi.2011.0118. PMC3339379 22455463

[149] Bhuva D. D., Smyth G. K., Garnham A., 2023.

[150] Hadley. Wickham, J Stat Softw 2009, 35.

[151] Zhang Y., Liu T., Hu X., Wang M., Wang J., Zou B., Tan P., Cui T., Dou Y., Ning L., Huang Y., Rao S., Wang D., Zhao X., Nucleic Acids Res 2021, 49, 8520. 34331449 10.1093/nar/gkab638 PMC8421219

